# Simultaneous Analysis of Thirteen Compounds in Yeokwisan Using High-Performance Liquid Chromatography–Photodiode Array Detection and Ultra-Performance Liquid Chromatography–Tandem Mass Spectrometry and Their Antioxidant Effects

**DOI:** 10.3390/ph17060727

**Published:** 2024-06-04

**Authors:** Chang-Seob Seo, So-Yeon Kim, Dong-Seon Kim

**Affiliations:** KM Science Research Division, Korea Institute of Oriental Medicine, Daejeon 34054, Republic of Korea; csseo0914@kiom.re.kr (C.-S.S.); kimsy0913@kiom.re.kr (S.-Y.K.)

**Keywords:** simultaneous analysis, Yeokwisan, HPLC–PDA, UPLC–MS/MS, antioxidant effect

## Abstract

Yeokwisan (YWS) is an herbal medicine prescription consisting of six oriental herbal medicines, developed to treat reflux esophagitis. We focused on developing an analytical method capable of simultaneously quantifying 13 compounds in YWS samples using high-performance liquid chromatography–photodiode array detection (HPLC–PDA) and ultra-performance liquid chromatography–tandem mass spectrometry (UPLC–MS/MS) and exploring their antioxidant effects. All compounds examined in both analytical systems were chromatographically separated on a SunFire^TM^ C_18_ (4.6 × 250 mm, 5 μm) column and an Acquity UPLC BEH C_18_ (2.1 × 100 mm, 1.7 μm) column using gradient elution of a water–acetonitrile mobile phase. Antioxidant effects were evaluated based on radical scavenging activity (DPPH and ABTS tests) and ferrous ion chelating activity. In two analytical methods, the coefficient of determination of the regression equation was ≥0.9965, the recovery range was 81.11–108.21% (relative standard deviation (RSD) ≤ 9.33%), and the precision was RSD ≤ 11.10%. Application of the optimized analysis conditions gave quantitative analysis results for YWS samples of 0.02–100.36 mg/g. Evaluation of the antioxidant effects revealed that baicalein and baicalin exhibit significant antioxidant activity, suggesting that they play an important role in the antioxidant effects of YWS.

## 1. Introduction

Herbal medicine prescriptions, which consist of combinations of at least two herbal medicines, have long been used for the treatment or prevention of diseases because of their multicomponent and multitarget characteristics and low side effects [1,2,3,4]. As a result, interest continues to grow to this day. Yeokwisan (YWS) is an herbal medicine prescription consisting of six oriental herbal medicines (Glycyrrhizae Radix et Rhizoma, Massa Medicata Fermentata, Phyllostachyos Caulis in Taeniam, Ponciri Fructus Immaturus, Scutellariae Radix, and Ostreae Testa) in a ratio of 3.3:1.7:1.0:3.5:9.0:1.3. YWS was developed at Chungju Weedahm Integrative Hospital (Chungju, Korea) for the clinical treatment of patients suffering from functional dyspepsia (FD) [5].

FD is one of the most common diseases of the digestive system; symptoms include abdominal distension, epigastric pain, early satiety, and postprandial fullness [6,7,8,9,10]. It varies slightly from country to country, it has a prevalence of around 16%, takes at least six months to diagnose, and has a significant impact on people’s quality of life and work efficiency [6,7,8,9,10].

Many studies have reported on ways to improve FD symptoms or develop treatments using traditional herbal medicines [11,12,13,14] or herbal medicine prescriptions [6,15,16,17]. Recently, Hwang et al. reported the effectiveness of YWS in improving gastric emptying through modulation of the ghrelin pathway in loperamide-induced FD using a mouse model [5]. The efficacy of some of the medicinal herbs of YWS (Massa Medicata Fermentata, Phyllostachyos Caulis in Taeniam, and Ponciri Fructus Immaturus) against FD has been reported [18,19,20]. However, no studies have yet been reported on the antioxidant effects of YWS.

The standardization of samples is essential for achieving the consistency of efficacy. In particular, it is increasingly required in herbal medicine prescriptions consisting of multiple herbal medicines. Their quality control is performed using analytical techniques such as high-performance liquid chromatography (HPLC) in conjunction with various modes of detection, including ultraviolet (UV), photodiode array, evaporative light scattering, mass spectrometry, and gas chromatography coupled with mass spectrometry [21,22,23,24]. In a previously reported study of YWS [5], six components were identified using a HPLC system (naringin, baicalin, poncirin, baicalein, glycyrrhizic acid, and wogonin). In addition to this—as far as we are aware—no analytical methods for the quality control of YWS have been reported.

Therefore, in this study, we attempted to develop a simultaneous analysis method for the quality control of YWS using 13 components (liquiritin apioside, liquiritin, 4-hydroxycinnamic acid, narirutin, naringin, ononin, baicalin, poncirin, wogonoside, baicalein, isoliquiritigenin, glycyrrhizin, and wogonin; Figure S1) in both HPLC–photodiode array detection (HPLC–PDA) and ultra-performance liquid chromatography–tandem mass spectrometry (UPLC–MS/MS) systems. Furthermore, the antioxidant effects of YWS and its major components were investigated, making use of the 2,2-diphenyl-1-picrylhydrazyl (DPPH) radical scavenging assay, 2-2′-azino-bis(3-ethylbenzothiazoline-6-sulfonic acid (ABTS) assay, and ferrous ion (Fe^2+^) chelating (FIC) activity.

## 2. Results and Discussion

### 2.1. Development of Simultaneous Analysis Method Using the HPLC–PDA System

#### 2.1.1. Selection of Marker Compounds for Development of the Simultaneous Analysis Method by HPLC–PDA

**Figure 1:**
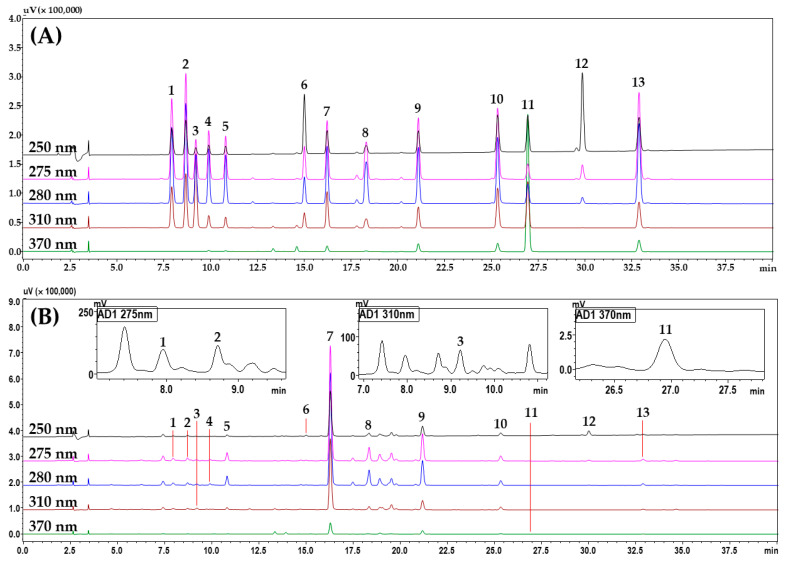
Representative HPLC chromatograms of the mixed standard solution (**A**) and YWS sample (**B**). Liquiritin apioside (1), liquiritin (2), 4-hydroxycinnamic acid (3), narirutin (4), naringin (5), ononin (6), baicalin (7), poncirin (8), wogonoside (9), baicalein (10), isoliquiritigenin (11), glycyrrhizin (12), and wogonin (13). The concentrations of each marker compound in the mixed standard solution were as follows: 4-hydroxycinnamic acid (10.00 μg/mL); ononin, baicalin, wogonoside, baicalein, isoliquiritigenin, and wogonin (20.00 μg/mL); narirutin and naringin (30.00 μg/mL); liquiritin (40.00 μg/mL); liquiritin apioside and poncirin (50.00 μg/mL); and glycyrrhizin (100.00 μg/mL).

To select markers for the quality control of YWS, the main ingredients of each herbal medicine that constitutes YWS were investigated. A total of 23 ingredients were selected as candidate marker compounds. Thus, the main ingredients investigated from each herbal medicine component were the following: liquiritin, liquiritin apioside, isoliquiritin, ononin, liquiritigenin, isoliquiritigenin, and glycyrrhizin from Glycyrrhizae Radix et Rhizoma; ferulic acid and γ-oryzanol from Massa Medicata Fermentata; chlorogenic acid, caffeic acid, isoorientin, 4-hydroxybenzaldehyde, 4-hydroxycinnamic acid, and vanillin from Phyllostachyos Caulis in Taeniam; narirutin, naringin, neoponcirin, and poncirin from Ponciri Fructus Immaturus; and baicalin, wogonoside, baicalein, and wogonin from Scutellariae Radix. The main ingredient of Ostreae Testa is calcium carbonate, which was excluded as a candidate. Samples of each herbal medicine constituting YWS and candidate ingredients were compared, and a comparison of the HPLC chromatograms is shown in Figure S2. According to the comparison results, 13 compounds out of the 23 candidate ingredients were finally detected in the YWS sample (Figure 1). These were selected as marker compounds for the quality control of YWS using HPLC–PDA, and a method for simultaneous analysis was subsequently developed. The peak in the sample was confirmed by comparing the retention time and UV spectrum of the standard compound.

#### 2.1.2. Optimization of HPLC Conditions for Development of the Simultaneous Analysis Method

Various parameters (e.g., column type, column temperature, type of acid, and flow rate) were explored in efforts to develop an optimal HPLC analysis method for the simultaneous analysis of 13 selected marker compounds from YWS.

As a first attempt, we compared reverse-phase columns from different manufacturers, e.g., the Waters SunFire^TM^ (Milford, MA, USA), Shiseido Capcell Pak UG80 (Tokyo, Japan), YMC Korea YMC-Triart (Seongnam, Republic of Korea), and Thermo Fisher Scientific Hypersil GOLD (San Jose, CA, USA). Their inner diameters, lengths, and particle sizes were all the same: 4.6 mm, 250 mm, and 5 μm, respectively. The results recorded for the various columns (Figure S3) revealed that the 13 marker compounds were well separated on the Waters SunFire^TM^ column (Figure S3A). However, with the Capcell Pak UG80 column (Figure S3B) and Hypersil GOLD column (Figure S3D), the separation of baicalin and ononin was not achieved. Furthermore, for the YMC-Triart column (Figure S3C), isoliquiritigenin and glycyrrhizin were detected at the same retention time, and the resolution of poncirin was not as good as that achieved with the other columns. Although the resolution of 4-hydroxycinnamic acid on the SunFire^TM^ column was slightly low, we were confident that it would be possible to resolve the issue by using other analysis conditions (e.g., by changing the temperature of the column and the type of acid added to the mobile phase). Considering all other marker compounds, the SunFire^TM^ column was then selected for subsequent simultaneous analysis.

In the next step, after column selection, we compared column temperatures (35, 40, 45, and 50 °C; Figure S4). At 35 °C (Figure S4A), the 13 marker compounds were well separated without interference from neighboring peaks, but complete separation was not achieved at 40, 45, and 50 °C. In the chromatogram at 40 °C (Figure S4B), 4-hydroxycinnamic acid was not completely separated from neighboring peaks, and at 45 °C the peaks of liquiritin apioside and 4-hydroxycinnamic acid had a poor resolution from neighboring peaks (Figure S4C). Furthermore, at 50 °C, the overlapping of liquiritin apioside with an unknown peak was detected and the resolution of liquiritin was not good (Figure S4D). Taking all the above results into consideration, the preferred temperature of the column was then established to be 35 °C.

As a third step, HPLC chromatograms were compared according to the type of acid added to the water–acetonitrile mobile phase system (formic acid, trifluoroacetic acid, and acetic acid) on the column and at the column temperature determined in the first and second steps. It is evident from Figure S5 that when 0.1% trifluoroacetic acid and 1.0% acetic acid were added, the resolutions of ononin, baicalin, and poncirin were significantly lower than those achieved with 0.1% formic acid. Hence, we selected to use 0.1% formic acid as the acid of choice to be added to the mobile phase system.

As a final step in the method optimization, flow rates were compared. There were no significant differences between using 0.8 mL/min and 1.0 mL/min (Figure S6).

Subsequently, after combining and comparing the various parameters discussed above, the optimal HPLC conditions for the simultaneous analysis of 13 marker compounds in a YWS sample were the following: Waters SunFire^TM^ column, column temperature 35 °C, addition of 0.1% formic acid, and flow rate 1.0 mL/min. Table S1 details the gradient elution conditions of the mobile phase and other parameters. Using the established optimal analysis conditions, all markers were completely eluted within 35 min with a resolution of >2.5, without interference from neighboring peaks. The chromatogram measured under the finally established HPLC analytical conditions is shown in Figure 1.

#### 2.1.3. Method Validation of the Developed HPLC Analysis Assay

Various method validation factors were evaluated for the HPLC assay optimized for the simultaneous analysis of the 13 marker compounds in YWS. The peaks were evaluated using several parameters, such as the retention factor (*k*′, 2.10–11.87), selectivity factor (*α*, 1.07–1.51), theoretical plate numbers per meter (*N*/m, 69,811–971,019), resolution (*Rs*, 2.51–21.35), and symmetry factor (*S*, 0.96–1.17) (Table S2), and all parameters in the developed HPLC assay showed satisfactory results within the acceptance criteria [25,26]. The range of the coefficient of determination (*r*^2^) value in the calibration curve of each marker compound prepared at different concentrations was 0.9999–1.0000, indicating a very good linearity (Table 1). The concentrations of the limit of detection (LOD) and limit of quantitation (LOQ) for evaluating sensitivity were calculated as 0.01–0.28 μg/mL and 0.04–0.85 μg/mL, respectively (Table 1 and Figure S7). The signal-to-noise ratio in the LOD and LOQ chromatograms was ≥3 and ≥10, respectively. In this analysis method, the recovery of each marker compound was 95.20–105.52% (RSD ≤ 2.64%, Table 2), indicating satisfactory results within the acceptable range of ±20%. Precision, assessed by the RSD value for each marker, was <2.01%, well within the acceptable range of ≤20% (Table 3 and Table S3).

#### 2.1.4. Simultaneous Determination of 13 Marker Compounds in a YWS Sample by the HPLC–PDA Assay

The newly developed and validated HPLC–PDA assay was satisfactorily applied for the simultaneous determination of the 13 marker compounds (liquiritin apioside, liquiritin, 4-hydroxycinnamic acid, narirutin, naringin, ononin, baicalin, poncirin, wogonoside, baicalein, isoliquiritigenin, glycyrrhizin, and wogonin) from a YWS sample. As performed for the detection wavelength shown in Table 1, quantification was performed by selecting the maximum UV absorption wavelength of each marker compound using a PDA detector. Under this assay, 13 marker compounds were detected at 0.02–99.03 mg per g of freeze-dried YWS (Table 4). Among them, baicalin (the main ingredient of Scutellariae Radix) was detected most abundantly, with a content of 99.03 mg/g. Poncirin, glycyrrhizin, wogonoside, and naringin were also detected in relatively abundant quantities compared with other marker compounds.

### 2.2. Development of Simultaneous Analysis Method Using the UPLC–MS/MS System

#### 2.2.1. Conditions for UPLC–MS/MS Multiple Reaction Monitoring (MRM) Method for Simultaneous Analysis of the 13 Marker Compounds in a YWS Sample

The development of the UPLC–MS/MS MRM analytical method was performed for the quality control of YWS using the 13 marker compounds selected earlier for the HPLC–PDA analysis method. These markers were detected using an electrospray ionization source. All of the marker compounds were compared in negative and positive ion modes to optimize mass conditions. Depending on the polarity of each compound, the sensitivity of 3 of the markers (liquiritin apioside, liquiritin, and 4-hydroxycinnamic acid) was better under negative ion mode, while that of the other 10 marker compounds (narirutin, naringin, ononin, baicalin, poncirin, wogonoside, baicalein, isoliquiritigenin, glycyrrhizin, and wogonin) was higher under positive ion mode.

**Figure 2:**
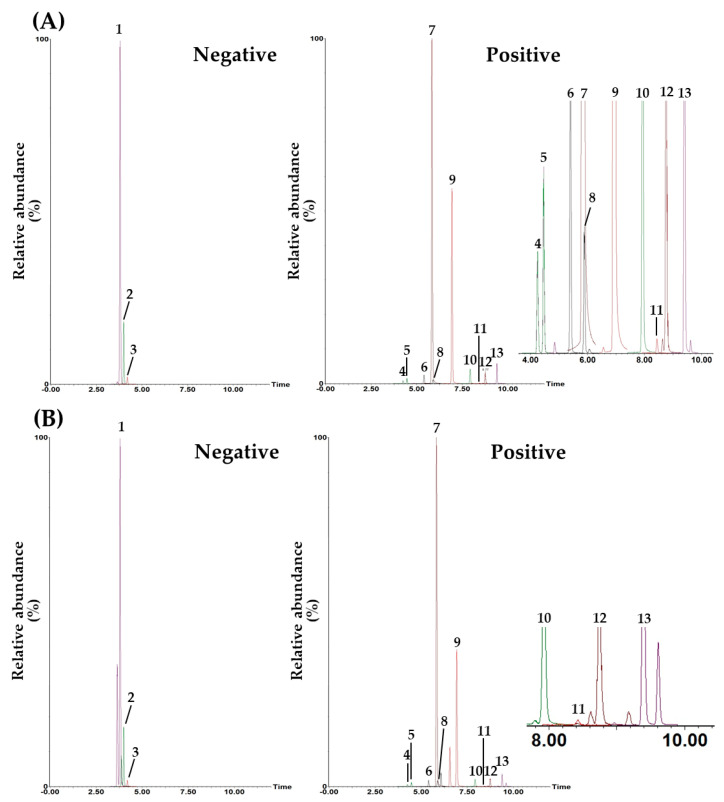
Total ion chromatograms of the mixed standard solution (**A**) and YWS sample (**B**) measured by the established UPLC-MS/MS MRM method. Liquiritin apioside (1), liquiritin (2), 4-hydroxycinnamic acid (3), narirutin (4), naringin (5), ononin (6), baicalin (7), poncirin (8), wogonoside (9), baicalein (10), isoliquiritigenin (11), glycyrrhizin (12), and wogonin (13). The concentrations of the 13 marker compounds in the mixed standard solution were as follows: 12 μg/L (isoliquiritigenin), 280 μg/L (liquiritin and ononin), 300 μg/L (4-hydroxycinnamic acid), 520 μg/L (wogonin), 1040 μg/L (nar-irutin), 1080 μg/L (poncirin), 1400 μg/L (baicalein), 2320 μg/L (isoliquiritin apioside), 4800 μg/L (naringin), 7600 μg/L (wogonoside), 8000 μg/L (glycyrrhizin), and 28,000 μg/L (baicalin).

Table 5 summarizes the optimal MRM conditions (precursor ion (Q1), product ion (Q3), cone voltage, and collision energy) for each marker compound established in this assay. That is, the MRM conditions for each marker in the YWS sample were set by comparing them to the standard compound. For liquiritin, ononin, baicalin, and wogonoside, each Q3 peak was set at *m*/*z* 254.99, 269.04, 271.00, and 285.03, in the form in which the glucose group was removed [27,28,29]. For liquiritin apioside and glycyrrhizin, Q1 peaks were observed at *m*/*z* 549.01 and 823.24, and *m*/*z* 255.02 and 453.18, generated by the removal of Api-Glc and 2Glc-H_2_O molecules, and they were set as Q3, respectively [30,31]. Other flavonoids, narirutin, naringin, and poncirin, were all designated as Q3 peaks at *m*/*z* 273.04, 273.04, and 287.03, generated by the Rha-Glc group leaving the Q1 peak, respectively [32,33]. For 4-hydroxycinnamic acid, isoliquiritigenin, and wogonin, carboxyl, C_8_H_7_O, and methyl molecules were removed from each Q1 peak, and the peaks observed at *m*/*z* 119.39, 137.01, and 269.97 were set as the Q3 peak, respectively [27,34]. Lastly, in the case of baicalein, the peak observed at *m*/*z* 123.00 due to retro-Diels–Alder cleavage at the C-ring in the structure was set as a Q3 peak [29]. Their fragmentations are shown in Figures S8 and S9. The total ion chromatogram of the mixed standard solution and YWS sample measured using optimal MRM conditions is shown in Figure 2. In addition, the extracted ion chromatograms are shown in Figure S10.

#### 2.2.2. Method Validation of the Developed UPLC–MS/MS Analysis Assay

In the analysis method by UPLC–MS/MS, the results for several parameters used as method validation for each marker are presented in Table 6, Table 7 and Table 8 and Table S7. For the *r*^2^ value, evaluating linearity and using the calibration curve, all markers showed a good linearity at ≥0.9965, and the LOD and LOQ concentrations that evaluated sensitivity were calculated as 0.003–4.60 μg/L and 0.01–13.81 μg/L, respectively (Table 6). The recovery was evaluated to be 81.11%–100.88% (RSD < 10%), which is suitable for the acceptable range of ±20% (Table 7). Lastly, the RSD value, which is a measure of precision evaluation, was 0.15%–11.10%; it was within the acceptable range (<20%) (Table 8 and Table S9). Taking all the above results into consideration, we demonstrated that the analytical method for the quality control of YWS using the UPLC–MS/MS system was appropriately developed.

#### 2.2.3. Quantification of the 13 Marker Components in a YWS Sample by the Developed UPLC–MS/MS MRM Analysis Method

The concentrations of the 13 marker compounds measured in a YWS sample by the UPLC–MS/MS MRM assay were 0.02–100.36 mg/g (Table 4). Similar to the HPLC–PDA assay results, baicalin (the major constituent of Scutellariae Radix) was detected most abundantly at 100.36 mg/g in the quantitative analysis by UPLC–MS/MS MRM assay. Furthermore, wogonoside, glycyrrhizin, naringin, and poncirin were present in a greater abundance than other ingredients in the YWS sample, and they showed a similar pattern to the HPLC–PDA analysis results.

### 2.3. Total Polyphenol and Total Flavonoid Contents

**Figure 3:**
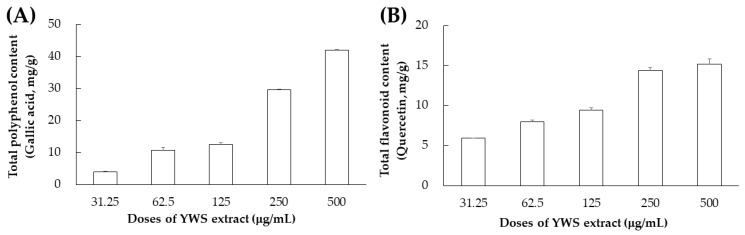
Total polyphenol (**A**) and flavonoid (**B**) contents of a YWS sample.

Total polyphenol content is reported to be a very important factor in determining the antioxidant capacity of foods [35,36]. Flavonoids are reported to be abundantly contained in the flowers, stems, and fruits of plants, and they are known to possess various functionalities including antioxidant, anticancer, and anti-inflammatory effects [37]. Upon measuring these two important factors that determine antioxidant capacity, it was confirmed that the YWS extract exhibits high polyphenol and flavonoid contents in a concentration-dependent manner (Figure 3).

### 2.4. Antioxidant Effects of YWS Sample and Its Marker Compounds

**Figure 4:**
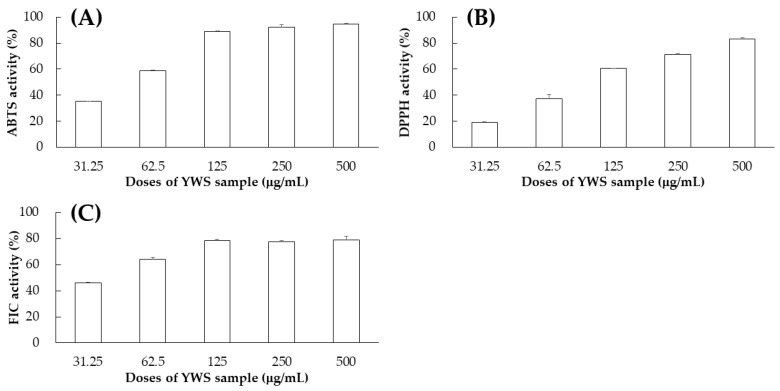
Effect of the YWS sample on free radical scavenging activity: ABTS (**A**), DPPH (**B**), and FIC (**C**) activities. Bars represent the standard deviation from duplicate determinations of each concentration.

We evaluated the antioxidant efficacy of YWS using DPPH (CAS No. 1898-66-4, Catalog No. D9132, Merck, Darmstadt, Germany), ABTS (CAS No. 30931-67-0, Catalog No. A1888, Merck, Darmstadt, Germany), and FIC assays. The results revealed high antioxidant activities across all three assays, indicating that YWS possesses strong capabilities to scavenge free radicals and chelate iron (Figure 4). To identify the active components responsible for this antioxidant activity, we selected 13 marker compounds from YWS. The evaluation of these isolated compounds revealed that some of them exhibited significant antioxidant activity (Table 9). Among them, baicalein and baicalin displayed the strongest antioxidant activity. Baicalein displayed exceptionally low IC_50_ values of 14.17 ± 2.17 μM for DPPH and 18.53 ± 0.6 μM for ABTS, along with a notable IC_50_ of 31.3 ± 2.5 μM in the FIC assay, underscoring its strong radical scavenging and iron-chelating abilities. Baicalin also displayed considerable antioxidant efficacy, with IC_50_ values of 48.91 ± 2.37 μM for DPPH, 33.03 ± 0.91 μM for ABTS, and 58.2 ± 0.3 μM for FIC. The compound 4-hydroxycinnamic acid displayed moderate efficacy in DPPH scavenging at 79.27 ± 3.86 μM, although it was more effective in the ABTS assay with an IC_50_ of 49.51 ± 0.72 μM. For the remaining single compounds, none of the three activity evaluations showed efficacy. The investigation revealed that the effects of YWS are largely attributed to specific isolated compounds, particularly to baicalein and baicalin, which demonstrated the highest efficacy in scavenging free radicals and chelating iron ions.

## 3. Materials and Methods

### 3.1. Plant Materials

The six raw herbal medicines (Table S4), five plant-derived herbal medicines, and an animal-derived herbal medicine were purchased from Kwangmyungdang Pharmaceutical (Ulsan, Republic of Korea). Prior to use, they were subjected to morphological sensory tests by Dr. Goya Choi, Korea Institute of Oriental Medicine (KIOM, Daejeon, Republic of Korea). Among the five plant-derived herbal medicines, the scientific names of four herbal medicines were confirmed by World Flora Online Plant List (www.wfoplantlist.org, accessed on 1 May 2024) [38]. Specimens (YWS–1 to YWS–6) of each herbal medicine in YWS were stored in the Korean Medicine Science Research Division, KIOM.

### 3.2. Chemicals and Reagents

The 13 reference standards selected as marker compounds for the quality control of YWS were purchased from natural product manufacturing supply companies: Shanghai Sunny Biotech (Shanghai, China), ChemFaces Biochemical (Wuhan, China), Biopurify Phytochemicals (Chengdu, China), Fujifilm Wako Pure Chemicals (Osaka, Japan), and ChemNorm Biotech (Wuhan, China). Table S5 and Figure S1 provide detailed information and the chemical structures, respectively, of the marker compounds analyzed. The organic solvents (methanol, ethanol, and acetonitrile) and acids (formic acid, trifluoroacetic acid, and acetic acid) added to the mobile phase system and used for analytical method development were either of HPLC or LC–MS grade. They were purchased from JT Baker (Phillipsburg, NJ, USA), Merck (Darmstadt, Germany), or Thermo Fisher Scientific (Cleveland, OH, USA). Ultrapure deionized water (15.0 MΩ·cm) was obtained from an Elix Technology Inside system (Milli-Q Integral 15, Merck, Millipore, France).

### 3.3. Preparation of YWS Sample

A YWS sample was prepared by mixing according to the quantities listed in Table S4. Among the six herbal medicines that constitute YWS, five herbal medicines (each 450 g) were extracted with 30% ethanol, while Ostreae Testa (1200 g) was extracted with water. This process was performed using the reflux extraction method. The organic solvent was removed from the extracted solution (of the extract) using a rotary evaporator. The residue was suspended by adding distilled water, followed by freeze drying, to afford the respective powdered samples. Each sample thus prepared was then used according to the ratio given in Table S4 to prepare a YWS sample; the prepared sample was stored at −20 °C until required for use.

### 3.4. Instrumentation and Analytical Conditions for Development of the HPLC–PDA Assay

The Prominence LC-20A series HPLC system (Shimadzu, Kyoto, Japan) was used for the simultaneous analysis of 13 marker compounds in YWS. The system combines a solvent delivery system, column oven, online degasser, autosampler, and PDA detector and is controlled by LC solution software (version 5.53). The 13 markers were separated on a Waters SunFire^TM^ column (250 × 4.6 mm, 5 μm; Waters, Milford, MA, USA) using a water–acetonitrile mobile phase gradient elution system (both containing 0.1% (*v*/*v*) formic acid). Table S1 summarizes the detailed HPLC operating conditions.

For the simultaneous analysis of 13 marker compounds in YWS, 100 mg of prepared YWS sample was dissolved in 10 mL of 70% methanol and then subjected to ultrasonic extraction in a Branson 8810 ultrasonic bath (Branson Ultrasonics, Denbury, CT, USA) for 1 h at room temperature. The extract was filtered through a GVS Abluo 0.2 μm syringe filter (diameter 25 mm; Zola Predosa, Italy) and then injected into the HPLC instrument. The quantification of some of the compounds involved their tenfold dilution, using 70% methanol, prior to analysis.

### 3.5. Instrumentation and Analytical Conditions for Development of the UPLC–MS/MS Assay

The MRM quantitative analysis of the 13 marker compounds in a YWS sample was carried out using an UPLC–MS/MS system comprising a Waters Acquity UPLC H-Class system and a TQ-S micro MS system (Milford, MA, USA). Detailed operational information of UPLC–MS/MS is provided in Table S6. Various parameters for the qualitative and quantitative analysis of the target by the UPLC–MS/MS MRM assay are given in Table 5. These included the ion mode and MRM transition (including cone voltage and collision energy) for qualitative and quantitative analyses.

To simultaneously analyze 13 marker compounds in YWS using the UPLC–MS/MS system, 308.5 mg of prepared YWS sample was dissolved in 100 mL of 70% methanol. The solution was subjected to ultrasonic extraction (10.0 min) and vortexing (10.0 min). The extract was filtered through a 0.2 μm syringe filter and then injected into the UPLC–MS/MS system.

### 3.6. Method Validation of Optimized Assays in HPLC–PDA and UPLC–MS/MS Systems

The system suitability test in the developed HPLC assay was evaluated based on various parameters such as *k*′, *α*, *N*, *Rs*, and *S*. The acceptable ranges for these parameters were determined to be *k*′ > 2.0, *α* > 1.0, *N* > 2000, *Rs* > 1.5, and *S* < 2.0, respectively [26,27]. In addition, to validate the two analytical methods that we developed, various factors were evaluated, including linearity, sensitivity, accuracy, and precision. The linearity of both analytical methods was assessed by the *r*^2^ value in the regression equation for each analyte. For the HPLC–PDA method, the LOD and LOQ values to evaluate the sensitivity of each marker compound were calculated using the regression equation of the calibration curve with the following equation:

$$LOD=3.3\times \frac{\sigma }{S}\;\mathrm{and}\;LOQ=10\times \frac{\sigma }{S}$$

where *σ* is the standard deviation (SD) of the *y*-intercept and *S* is the slope of the regression equation.

Meanwhile, in the case of the UPLC–MS/MS method, the LOD and LOQ values were calculated using signal-to-noise ratios of 3:1 and 10:1, respectively.

The recovery was evaluated using a standard addition method. Three known concentrations (low, medium, and high) of each marker compound were added to the YWS sample, and extraction was carried out according to the methods given in Section 3.4 and Section 3.5, followed by analysis by the HPLC–PDA and UPLC–MS/MS methods, respectively. The acceptable limit for recovery was set at ±20%. The recovery was calculated using the following equation:

$$Recovery\left(\%\right)=\frac{found\;amount}{spiked\;amount}\times 100$$

Precision was demonstrated by the RSD value of each marker after measuring for three different concentrations (low, medium, and high) over a period of one day and over three consecutive days. In addition, the repeatability of each marker was evaluated based on the RSD values of retention time and peak area after measuring the standard solution six times. The acceptable range of precision testing was set to ≤20%. RSD (%) was calculated using the following equation:

$$RSD\left(\%\right)=\frac{SD}{Mean}\times 100$$

### 3.7. Total Polyphenol and Total Flavonoid Contents

The total polyphenolic compound content was measured by a modified Folin–Denis method [39]. A 100 μL volume of 2 N Folin–Ciocalteu reagent was added to 100 μL of the sample, followed by the addition of 500 μL of a 10% sodium carbonate solution, then mixing. Distilled water (800 μL) was added to the mixture. The reaction proceeded at room temperature for 1 h, whereafter the absorbance was measured at 750 nm. At this point, gallic acid was used as a standard material, and the total polyphenolic compound content was measured from the standard curve obtained after analysis in the same manner as for the sample.

The total flavonoid content was measured using the Moreno method [40]. To 500 μL of the sample, 0.1 mL each of 10% aluminum nitrate and 1 M potassium acetate, and then 4.3 mL of ethanol, were sequentially added. After mixing, the resulting solution was held at room temperature for 40 min. The absorbance was measured at 415 nm using a microplate reader. The total flavonoid content of the sample was measured from a standard calibration curve obtained using quercetin (CAS No. 117-39-5, Catalog No. PHL89262, Merck, Darmstadt, Germany) as a standard material, and the result was calculated as the equivalent amount of quercetin (μg/mL).

### 3.8. Antioxidant Effects of YWS Sample and Its Marker Compounds

#### 3.8.1. DPPH Radical Scavenging Assay

The DPPH radical scavenging ability means the degree to which the DPPH radical is reduced by this reaction as a result of the effect of electron donation within the sample on the stable free radical DPPH. The activity was used by partially modifying the method of Blois [41]. A 0.15 mM DPPH solution and each concentration sample were mixed, reacted in the dark for 30 min, and then the absorbance was measured at 517 nm using a microplate reader (SpectraMax M2, Molecular Devices, Sunnyvale, CA, USA). Ascorbic acid (CAS No. 50-81-7, Catalog No. 47863, Merck, Darmstadt, Germany) was used as a positive control. DPPH radical scavenging activity was calculated according to the following equation:

$$DPPH\;radical\;scavenging\;activity\left(\%\right)=1-\frac{Abs}{Abc}\times 100$$

where *Abs* is the absorbance of sample and *Abc* is the absorbance of the control.

#### 3.8.2. ABTS Activity

The ABTS (CAS No. 30931-67-0, Catalog No. A1888, Merck, Darmstadt, Germany) radical scavenging ability was measured by partially modifying the method of Re et al. [42]. ABTS free radicals are produced by a chemical oxidation reaction with potassium persulfate. ABTS was diluted in ethanol to prepare a 7 mM solution. It was mixed with 2.4 mM potassium persulfate solution (1:1) and then stored in the dark at room temperature for 12 h to produce radicals. The absorbance of the mixed ABTS solution was measured at 734 nm. The solution was then diluted with ethanol until the absorbance reached 0.7 ± 0.05—this was then used in the experiment. After adding 180 μL of ABTS solution to 20 μL of each sample concentration and reacting at room temperature for 10 min, the absorbance was measured at 734 nm using a microplate reader. Ascorbic acid was used as a positive control. ABTS radical scavenging activity was calculated according to the following equation:

$$ABTS\;radical\;scavenging\;activity\left(\%\right)=1-\frac{Abs}{Abc}\times 100$$

where *Abs* is the absorbance of the sample and *Abc* is the absorbance of the control.

#### 3.8.3. FIC Activity

FIC was measured using an FIC assay kit from Zen-Bio (Durham, NC, USA). Each sample was subjected to a ferrozine working solution, left at room temperature for 10 min, and then the absorbance was measured at 562 nm. Ethylenediamine tetraacetic acid (EDTA, CAS No. 60-00-4, Catalog No. E9884, Merck, Darmstadt, Germany) was used as a positive control. Activity was calculated using the following equation:

$$Ferrous\;ion\;chelating\left(\%\right)=\frac{Absmax-Abstest}{Absmax}\times 100$$

where *Absmax* is the maximal absorbance value by iron sulfate, ferrozine, and the assay buffer, and *Abstest* is the absorbance value of the sample.

### 3.9. Statistical Analysis

All experiments were performed in triplicate, and the measurements are presented as the mean ± standard deviation. Statistical significance between experimental groups was analyzed using Student’s two-tailed t-test. Statistical significance was judged at *p* < 0.05.

## 4. Conclusions

Herein, to control the quality of YWS developed for the clinical treatment of patients with FD, simultaneous HPLC–PDA and UPLC–MS/MS MRM analysis methods were developed and the antioxidant effects of 13 compounds that YWS contains were explored. The developed methods were validated using analytical parameters such as linearity, sensitivity, accuracy, and precision. In addition, antioxidant effects were considered through DPPH, ABTS, and FIC efficacy evaluation. Our knowledge established here can be used as basic data for conducting clinical studies or other efficacy studies, as well as for the quality control of YWS—a newly established herbal medicine prescription.

## Figures and Tables

**Table 1:** Wavelength for quantification, linear range, regression equation, coefficient of determination (*r*^2^), limit of detection (LOD), and limit of quantitation (LOQ) using HPLC–PDA simultaneous analysis of the 13 marker compounds in YWS.

Analyte ^1^	Detected Wavelength (nm)	Linear Range (μg/mL)	$\mathrm{Regression}\;\mathrm{Equation}{\;}^{2}\;y=ax+b$	*r* ^2^	LOD (μg/mL)	LOQ (μg/mL)
1	275	0.31–20.00	*y* = 18,211.69*x* + 1008.30	1.0000	0.04	0.11
2	275	0.31–20.00	*y* = 28,044.42*x* + 1604.28	1.0000	0.01	0.04
3	310	0.31–20.00	*y* = 100,148.87*x* + 5544.60	1.0000	0.03	0.08
4	280	0.78–50.00	*y* = 28,290.54*x* + 4655.94	0.9999	0.06	0.17
5	280	0.47–30.00	*y* = 18,281.87*x* + 1530.74	1.0000	0.03	0.09
6	250	0.47–30.00	*y* = 36,397.22*x* + 2960.46	1.0000	0.04	0.12
7	275	2.34–150.00	*y* = 36,882.40*x* + 12,832.74	1.0000	0.28	0.85
8	280	0.78–50.00	*y* = 18,071.54*x* + 2503.17	1.0000	0.07	0.21
9	275	0.47–30.00	*y* = 58,815.90*x* + 4399.17	1.0000	0.04	0.12
10	275	0.78–50.00	*y* = 65,548.02*x* + 83,066.84	0.9999	0.09	0.28
11	370	0.31–20.00	*y* = 73,467.19*x* + 3382.49	1.0000	0.02	0.08
12	250	0.78–50.00	*y* = 8533.07*x* + 731.10	1.0000	0.04	0.13
13	275	0.31–20.00	*y* = 84,077.35*x* + 4664.54	1.0000	0.02	0.08

^1^ Liquiritin apioside (1), liquiritin (2), 4-hydroxycinnamic acid (3), narirutin (4), naringin (5), ononin (6), baicalin (7), poncirin (8), wogonoside (9), baicalein (10), isoliquiritigenin (11), glycyrrhizin (12), and wogonin (13). ^2^ *y*: peak area of compounds; *x*: concentration (μg/mL) of compounds.

**Table 2:** Recovery (%) of the 13 marker compounds in the developed HPLC–PDA assay.

Analyte ^1^	Original Amount (μg/mL)	Spiked Amount (μg/mL)	Found Amount (μg/mL)	Recovery (%)	SD ^2^	RSD ^3^ (%)
1	6.93	1.00	7.96	103.62	2.02	1.95
		3.00	9.87	98.24	0.79	0.81
		6.00	12.73	96.68	0.60	0.62
2	4.13	1.00	5.13	99.49	2.51	2.53
		2.00	6.14	100.56	2.21	2.19
		4.00	8.10	99.21	1.33	1.35
3	6.44	1.00	7.45	101.14	2.66	2.63
		3.00	9.44	99.81	1.06	1.06
		6.00	12.20	96.01	0.33	0.34
4	2.31	1.00	3.33	101.95	2.00	1.96
		2.00	4.42	105.31	0.71	0.68
		4.00	6.43	102.94	0.39	0.38
5	18.44	3.00	21.47	101.06	2.16	2.14
		7.50	25.59	95.26	0.91	0.95
		15.00	32.89	96.29	0.19	0.19
6	0.94	1.00	1.91	97.50	0.86	0.88
		2.00	3.05	105.52	0.97	0.92
		4.00	5.09	103.83	0.54	0.52
7	8.94	2.00	11.02	103.86	2.37	2.29
		5.00	13.96	100.22	0.47	0.47
		10.00	18.67	97.22	0.49	0.50
8	31.81	6.00	37.83	100.39	0.76	0.75
		15.00	46.54	98.20	0.34	0.34
		30.00	61.08	97.58	0.18	0.19
9	15.29	3.00	18.25	98.71	0.43	0.44
		7.50	22.43	95.20	0.06	0.06
		15.00	29.76	96.47	0.13	0.14
10	26.22	4.00	30.19	99.20	2.62	2.64
		10.00	36.10	98.81	2.07	2.09
		20.00	47.31	105.46	1.07	1.02
11	0.28	1.00	1.30	102.26	0.24	0.23
		2.00	2.39	105.38	0.23	0.22
		4.00	4.45	104.35	0.07	0.07
12	19.48	4.00	23.53	101.15	0.89	0.88
		10.00	29.13	96.50	0.20	0.20
		20.00	39.33	99.25	0.33	0.33
13	9.24	2.00	11.27	101.34	1.81	1.78
		5.00	14.33	101.69	1.12	1.10
		10.00	19.24	100.00	0.17	0.17

^1^ Information for all analytes is presented in a footnote to Table 1. ^2^ SD: standard deviation. ^3^ RSD: relative standard deviation.

**Table 3:** Precision of the 13 marker compounds in the developed HPLC–PDA assay.

Analyte ^1^	Conc. (μg/mL)	Intraday				Interday	
		Observed Conc. (μg/mL)	Precision (RSD, %)	Accuracy (%)	Observed Conc. (μg/mL)	Precision (RSD, %)	Accuracy (%)
1	5.0	5.11	0.50	102.27	5.21	1.79	104.23
	10.0	10.10	0.21	101.01	10.23	1.17	102.26
	20.0	20.13	0.52	100.66	20.51	1.89	102.56
2	5.0	5.11	0.42	102.28	5.21	1.80	104.26
	10.0	10.09	0.22	100.94	10.22	1.16	102.20
	20.0	20.11	0.57	100.57	20.49	1.87	102.44
3	5.0	5.11	0.43	102.17	5.20	1.77	104.09
	10.0	10.09	0.23	100.94	10.22	1.16	102.17
	20.0	20.09	0.58	100.47	20.47	1.84	102.34
4	12.5	12.81	0.52	102.46	13.09	2.01	104.72
	25.0	25.27	0.23	101.06	25.79	0.86	103.17
	50.0	50.28	0.66	100.56	51.27	1.97	102.54
5	7.5	7.68	0.44	102.34	7.82	1.76	104.32
	15.0	15.15	0.26	101.01	15.34	1.15	102.29
	30.0	30.17	0.59	100.58	30.74	1.86	102.48
6	7.5	7.66	0.43	102.20	7.81	1.75	104.11
	15.0	15.14	0.26	100.95	15.33	1.17	102.20
	30.0	30.15	0.64	100.51	30.71	1.84	102.36
7	37.5	38.34	0.41	102.24	39.05	1.74	104.12
	75.0	75.64	0.24	100.86	76.57	1.16	102.09
	150.0	150.76	0.63	100.51	153.51	1.82	102.34
8	5.0	5.11	0.50	102.27	5.21	1.92	104.27
	12.5	12.77	0.46	102.16	13.02	1.34	102.24
	25.0	25.23	0.26	100.92	25.57	1.95	102.37
9	50.0	50.34	0.65	100.68	51.29	1.79	104.12
	7.5	7.66	0.46	102.14	7.80	1.29	102.27
	15.0	15.12	0.25	100.81	15.31	1.92	102.58
10	30.0	30.18	0.64	100.59	30.73	1.75	104.03
	12.5	12.86	0.86	102.91	13.09	1.19	102.06
	25.0	25.34	0.24	101.36	25.64	1.83	102.44
11	50.0	50.41	0.56	100.82	51.28	1.74	104.74
	5.0	5.10	0.48	102.06	5.20	1.12	102.55
	10.0	10.08	0.24	100.79	10.21	1.74	102.56
12	20.0	20.10	0.64	100.52	20.48	1.78	103.98
	12.5	12.78	0.88	102.23	12.99	1.20	102.09
	25.0	25.11	0.32	100.43	25.43	1.85	102.39
13	50.0	49.97	0.69	99.93	50.91	1.71	103.93
	5.0	5.11	0.42	102.13	5.20	1.20	101.72
	10.0	10.10	0.27	101.00	10.22	1.89	101.83

^1^ Information for all analytes is presented in a footnote to Table 1.

**Table 4:** Concentration (mg/g) of 13 marker compounds in a YWS sample by HPLC–PDA and UPLC–MS/MS MRM assays.

Analyte ^1^	HPLC–PDA Assay			UPLC–MS/MS MRM Assay			Source ^2^
	Mean (mg/g)	SD	RSD (%)	Mean (mg/g)	SD	RSD (%)	
1	5.94	0.06	1.06	5.93	0.01	0.13	GRR
2	3.89	0.03	0.74	0.65	0.01	1.02	GRR
3	0.62	0.01 × 10^−2^	0.01	0.57	0.03	4.98	PCT
4	2.23	0.01	0.29	2.52	0.11	4.33	PFI
5	15.02	0.12	0.80	14.67	1.09	7.42	PFI
6	1.29	0.01 × 10^−1^	0.08	0.74	0.02	2.93	GRR
7	99.03	0.21	0.21	100.36	1.96	1.96	SR
8	27.32	0.05	0.19	6.94	0.17	2.41	PFI
9	15.96	0.04	0.23	19.90	0.19	0.93	SR
10	1.98	0.03 × 10^−1^	0.15	2.74	0.04 × 10^−2^	1.53	SR
11	0.02	0.02 × 10^−2^	0.67	0.02	0.04	1.82	GRR
12	18.31	0.10	0.55	19.35	0.33	1.72	GRR
13	0.95	0.03 × 10^−1^	0.27	1.20	0.01	1.11	SR

^1^ Information for all analytes is presented in a footnote to Table 1. ^2^ GRR: Glycyrrhizae Radix et Rhizoma; PCT: Phyllostachyos Caulis in Taeniam; PFI: Ponciri Fructus Immaturus; SR: Scutellariae Radix.

**Table 5:** Optimized parameters for UPLC–MS/MS MRM assay of the 13 marker compounds in YWS.

Analyte ^1^	Ion Mode	Exact Mass	MRM Transition		Cone Voltage (V)	Collision Energy (eV)
			Precursor Ion	Production Ion		
1	−	550.17	549.01	255.02	78	30
2	−	418.13	416.97	254.99	52	18
3	−	164.05	162.89	119.39	36	15
4	+	580.18	581.02	273.04	40	24
5	+	580.18	581.02	273.04	40	20
6	+	430.13	430.97	269.04	42	20
7	+	446.08	447.00	271.00	26	18
8	+	592.18	595.03	287.03	32	18
9	+	460.10	461.00	285.03	42	18
10	+	284.07	270.95	123.00	80	32
11	+	270.05	256.91	137.01	36	18
12	+	256.07	823.24	453.18	56	26
13	+	822.40	284.97	269.97	54	22

^1^ Information for all analytes is presented in a footnote to Table 1.

**Table 6:** Retention time, linear range, regression equation, *r*^2^, LOD, and LOQ values of the 13 marker compounds assessed by the UPLC–MS/MS MRM analytical assay.

Analyte ^1^	Retention Time (min)	Linear Range (μg/L)	$\mathrm{Regression}\;\mathrm{Equation}{\;}^{2}\;y=ax+b$	*r* ^2^	LOD (μg/L)	LOQ (μg/L)
1	3.80	145.00–2320.00	*y* = 117.84*x* − 98.42	0.9998	0.39	1.18
2	4.00	17.50–280.00	*y* = 188.23*x* − 336.34	0.9991	0.03×10^−1^	0.01
3	4.20	18.75–300.00	*y* = 31.85*x* − 11.87	0.9982	4.60	13.81
4	4.26	65.00–1040.00	*y* = 167.05*x* − 798.50	0.9977	0.29	0.87
5	4.47	300.00–4800.00	*y* = 73.74*x* − 2306.46	0.998	2.47	7.40
6	5.40	17.50–280.00	*y* = 5342.60*x* + 2610.58	0.9993	0.08	0.23
7	5.83	1750.00–28,000.00	*y* = 1920.04*x* + 1.27 × 10^6^	0.9965	0.80	2.39
8	5.89	67.50–1080.00	*y* = 222.63*x* + 7550.99	0.9971	0.19	0.57
9	6.92	475.00–7600.00	*y* = 3788.22*x* + 242,543.00	0.9991	0.01	0.02
10	7.92	87.50–1400.00	*y* = 1201.74*x* − 15,570.10	0.9989	0.27	0.81
11	8.43	0.75–12.00	*y* = 3636.80*x* − 160.47	0.9994	0.08	0.23
12	8.73	500.00–8000.00	*y* = 220.35*x* + 22,317.90	0.9972	3.50	10.51
13	9.39	32.50–520.00	*y* = 4444.41*x* + 17,728.90	0.9985	0.07	0.20

^1^ Information for all analytes is presented in a footnote to Table 1. ^2^ *y*: peak area of compounds; *x*: concentration (μg/L) of compounds.

**Table 7:** Recovery (%) of the 13 marker compounds assessed by the UPLC–MS/MS MRM analytical assay.

Analyte ^1^	Spiked Amount (μg/L)	Found Amount (μg/L)	Recovery (%)	SD	RSD (%)
1	120.00	117.68	98.07	8.07	1.14
	300.00	287.17	95.72	10.57	1.20
	600.00	638.11	106.35	114.80	9.33
2	14.00	13.15	93.93	1.48	1.90
	35.00	33.46	95.60	1.26	1.28
	70.00	67.61	96.59	1.41	1.06
3	14.00	15.15	108.21	4.46	6.16
	35.00	36.80	105.14	2.09	2.22
	70.00	70.65	100.93	2.45	1.92
4	50.00	43.83	87.66	25.13	8.45
	125.00	101.39	81.11	30.64	8.63
	250.00	253.36	101.34	44.33	8.74
5	200.00	206.45	103.23	41.90	2.52
	500.00	447.71	89.54	88.87	4.67
	1000.00	1080.73	108.07	54.58	2.15
6	14.00	15.11	107.93	3.79	4.25
	35.00	35.52	101.49	4.52	4.13
	70.00	72.06	102.94	4.12	2.82
7	1400.00	1506.24	107.59	320.34	2.77
	3500.00	3526.71	100.76	474.11	3.49
	7000.00	6826.44	97.52	718.95	4.26
8	60.00	61.41	102.35	35.12	4.64
	150.00	156.29	104.19	47.12	5.54
	300.00	251.27	83.76	88.21	9.32
9	400.00	385.35	96.34	33.51	1.41
	1000.00	936.03	93.60	52.04	1.78
	2000.00	1880.40	94.02	38.95	1.01
10	80.00	86.27	107.84	9.30	2.58
	200.00	188.89	94.45	12.71	2.75
	400.00	370.11	92.53	9.77	1.52
11	0.60	0.62	103.33	0.03	0.83
	1.50	1.49	99.33	0.09	2.39
	3.00	2.92	97.33	0.06	1.04
12	400.00	402.46	100.62	137.30	5.87
	1000.00	1058.19	105.82	203.91	6.81
	2000.00	2086.39	104.32	203.49	5.06
13	26.00	26.40	101.54	2.05	1.40
	65.00	64.93	99.89	4.24	2.29
	130.00	128.92	99.17	4.45	1.78

^1^ Information for all analytes is presented in a footnote to Table 1.

**Table 8:** Precision data of the 13 marker compounds assessed by the developed UPLC–MS/MS MRM analytical assay.

Analyte ^1^	Conc. (μg/L)	Intraday				Interday	
		Observed Conc. (μg/L)	Precision (RSD, %)	Accuracy (%)	Observed Conc. (μg/L)	Precision (RSD, %)	Accuracy (%)
1	145.00	144.49	2.06	99.65	146.51	1.37	101.04
	580.00	583.88	1.94	100.67	582.12	1.34	100.37
	2320.00	2313.55	0.63	99.72	2330.49	0.63	100.45
2	17.50	17.55	1.33	100.29	17.72	1.34	101.24
	70.00	70.40	1.51	100.58	70.23	1.93	100.33
	280.00	278.51	0.92	99.47	279.65	0.39	99.88
3	18.75	18.35	3.93	97.85	18.19	4.65	97.03
	75.00	76.06	2.84	101.41	73.46	3.34	97.94
	300.00	297.32	1.68	99.11	300.84	1.33	100.28
4	65.00	61.21	5.11	94.17	63.52	4.68	97.73
	260.00	277.62	7.50	106.78	269.33	6.26	103.59
	1040.00	1017.95	0.81	97.88	1027.21	2.32	98.77
5	300.00	297.86	5.16	99.29	302.65	2.02	100.88
	1200.00	1237.03	6.42	103.09	1216.11	4.04	101.34
	4800.00	4818.62	1.66	100.39	4907.27	1.99	102.23
6	17.50	16.77	1.07	95.81	16.78	0.59	95.87
	70.00	72.02	1.17	102.89	72.15	0.15	103.06
	280.00	275.91	0.51	98.54	279.81	2.06	99.93
7	1750.00	1601.09	5.55	91.49	1603.95	5.54	91.65
	7000.00	7481.61	2.87	106.88	7542.56	0.76	107.75
	28000.00	26816.89	0.88	95.77	26707.39	0.71	95.38
8	67.50	61.97	6.33	91.81	61.14	3.04	90.57
	270.00	291.57	7.12	107.99	289.07	4.43	107.06
	1080.00	1041.33	2.77	96.42	1051.48	1.00	97.36
9	475.00	453.76	1.24	95.53	458.77	1.43	96.58
	1900.00	1955.38	1.25	102.91	1924.62	1.50	101.30
	7600.00	7500.00	0.32	98.68	7546.88	0.55	99.30
10	87.50	91.19	1.71	104.21	92.36	1.41	105.55
	350.00	338.49	2.97	96.71	332.45	1.93	94.99
	1400.00	1401.20	2.05	100.09	1416.33	2.60	101.17
11	0.75	0.74	2.07	98.22	0.75	0.74	99.51
	3.00	3.01	0.51	100.22	2.98	0.67	99.33
	12.00	11.94	0.63	99.47	12.01	0.49	100.05
12	500.00	461.99	5.44	92.40	485.37	5.25	97.07
	2000.00	1976.57	7.17	98.83	2000.69	1.20	100.03
	8000.00	7780.40	4.44	97.26	7828.61	2.40	97.86
13	32.50	30.71	1.48	94.49	30.60	1.46	94.16
	130.00	134.47	0.58	103.44	133.63	0.57	102.79
	520.00	511.09	0.45	98.29	511.99	0.24	98.46

^1^ Information for all analytes is presented in a footnote to Table 1.

**Table 9:** Effects of 13 marker compounds on free radical scavenging activity.

Analyte ^1^	IC_50_ ^2^		
	DPPH	ABTS	FIC
1	>100	>100	>100
2	>100	>100	>100
3	79.27 ± 3.86	49.51 ± 0.72	>100
4	>100	>100	>100
5	>100	>100	>100
6	>100	>100	>100
7	48.91 ± 2.37	33.03 ± 0.91	58.2 ± 0.3
8	>100	>100	>100
9	>100	>100	>100
10	14.17 ± 2.17	18.53 ± 0.6	31.3 ± 2.5
11	>100	>100	>100
12	>100	>100	>100
13	>100	>100	>100
Ascorbic acid ^3^	5.24 ± 1.89	3.95 ± 1.26	-
EDTA ^4^	-	-	115.69 ± 1.95

Results are the mean ± standard deviation (n = 5). ^1^ Information for all analytes is presented in a footnote to Table 1. ^2^ Concentration (in μM) required for 50% reduction in activity. ^3^ Ascorbic acid was used as a positive control for DPPH and ABTS free radical scavenging activity. ^4^ EDTA was used as a positive control for FIC free radical scavenging activity.

## Data Availability

All data in this study can be found in this paper.

## Supplementary Materials

The following supporting information can be downloaded at: https://www.mdpi.com/article/10.3390/ph17060727/s1, Figure S1: Chemical structures of the 13 reference standard compounds selected as marker compounds for the quality control of YWS; Figure S2: HPLC–PDA chromatograms investigated for the selection of marker compounds: (A) blank sample; (B) mixed 23 standard compounds; (C) 70% methanol solution of lyophilized YWS sample; (D) Glycyrrhizae Radix et Rhizoma extract; (E) Massa Medicata Fermentata extract; (F) Phyllostachyos Caulis in Taeniam extract; (G) Ponciri Fructus Immaturus extract; (H) Scutellariae Radix extract; (I) Ostreae Testa extract. Chlorogenic acid (1), caffeic acid (2), isoorientin (3), 4-hydroxybenzaldehyde (4), 4-hydroxycinnamic acid (5), liquiritin apioside (6), vanillin (7), liquiritin (8), ferulic acid (9), narirutin (10), naringin (11), isoliquiritin (12), ononin (13), neoponcirin (14), liquiritigenin (15), poncirin (16), baicalin (17), wogonoside (18), baicalein (19), isoliquiritigenin (20), wogonin (21), glycyrrhizin (22), and γ-oryzanol (23); Figure S3: Comparison of HPLC–PDA chromatograms according to the column manufacturer. (A) SunFire^TM^ C_18_ column; (B) Capcell Pak UG80 C_18_ column; (C) YMC-Triart C_18_ column; (D) Hypersil GOLD C_18_ column. Liquiritin apioside (1), liquiritin (2), 4-hydroxycinnamic acid (3), narirutin (4), naringin (5), ononin (6), baicalin (7), poncirin (8), wogonoside (9), baicalein (10), isoliquiritigenin (11), glycyrrhizin (12), and wogonin (13); Figure S4: Comparison of HPLC–PDA chromatograms according to the column temperatures of the 13 selected marker compounds: 35 °C (A), 40 °C (B), 45 °C (C), and 50 °C (D). Liquiritin apioside (1), liquiritin (2), 4-hydroxycinnamic acid (3), narirutin (4), naringin (5), ononin (6), baicalin (7), poncirin (8), wogonoside (9), baicalein (10), isoliquiritigenin (11), glycyrrhizin (12), and wogonin (13); Figure S5: Comparison of HPLC–PDA chromatograms according to the type of acid for the 13 selected marker compounds: 0.1% (*v*/*v*) formic acid (A), 0.1% (*v*/*v*) trifluoroacetic acid (B), and 1.0% (*v*/*v*) acetic acid (C). Liquiritin apioside (1), liquiritin (2), 4-hydroxycinnamic acid (3), narirutin (4), naringin (5), ononin (6), baicalin (7), poncirin (8), wogonoside (9), baicalein (10), isoliquiritigenin (11), glycyrrhizin (12), and wogonin (13); Figure S6: Comparison of HPLC–PDA chromatograms according to the flow rate for the 13 selected marker compounds: 0.8 mL/min (A) and 1.0 mL/min (B). Liquiritin apioside (1), liquiritin (2), 4-hydroxycinnamic acid (3), narirutin (4), naringin (5), ononin (6), baicalin (7), poncirin (8), wogonoside (9), baicalein (10), isoliquiritigenin (11), glycyrrhizin (12), and wogonin (13); Figure S7: HPLC chromatograms at LOD (A) and LOQ (B) concentrations. Liquiritin apioside (1), liquiritin (2), 4-hydroxycinnamic acid (3), narirutin (4), naringin (5), ononin (6), baicalin (7), poncirin (8), wogonoside (9), baicalein (10), isoliquiritigenin (11), glycyrrhizin (12), and wogonin (13); Figure S8: Mass fragmentation of each marker compound for simultaneous analysis; Figure S9: Precursor ion (Q1; left) and product ion (Q3; right) peaks for each marker compound. Liquiritin apioside (1), liquiritin (2), 4-hydroxycinnamic acid (3), narirutin (4), naringin (5), ononin (6), baicalin (7), poncirin (8), wogonoside (9), baicalein (10), isoliquiritigenin (11), glycyrrhizin (12), and wogonin (13); Figure S10: Extracted ion chromatograms of each reference standard compound (A) and the 13 marker compounds selected in a YWS sample (B) by the UPLC–MS/MS MRM method in negative and positive ion modes; Table S1: Analytical conditions for simultaneous analysis of the nine target components in a YWS sample by HPLC–PDA; Table S2: System suitability for simultaneous analysis of the nine target components by HPLC–PDA; Table S3: Repeatability for retention time and peak area of the 13 marker compounds by HPLC–PDA assay; Table S4: Composition of and information on Yeokwisan; Table S5: Information on the 13 reference standard compounds selected as marker compounds for the quality control of YWS; Table S6: UPLC–MS/MS MRM conditions for simultaneous quantitative analysis of the 13 marker compounds in YWS sample; Table S7: Repeatability for retention time and peak area of the 13 marker compounds by UPLC–MS/MS MRM assay.
