# Supplementary material for: Simultaneous Analysis of Thirteen Compounds in Yeokwisan Using High-Performance Liquid Chromatography–Photodiode Array Detection and Ultra-Performance Liquid Chromatography–Tandem Mass Spectrometry and Their Antioxidant Effects
Source: Pharmaceuticals (Basel). 2024 Jun 4;17(6):727. doi: 10.3390/ph17060727 (PMC11206242; doi:10.3390/ph17060727)
Supplement: Supplementary file 1 [file pharmaceuticals-17-00727-s001.zip › pharmaceuticals-3024950-supplementary.pdf]

Table S1

Analytical conditions for simultaneous analysis of the nine target components in a YWS sample by HPLC–PDA.

Chromatographic parameter			
Column	SunFire™ C_{18} analytical column (250 mm × 4.6 mm, 5 μm)		
Detector	PDA (250, 275, 280, 310, and 370 nm)		
Flow rate	1.0 mL/min		
Injection volume	10.0 μL		
Column temperature	35.0 °C		
Mobile phase	A: 0.1% (v/v) formic acid in distilled water<br>B: 0.1% (v/v) formic acid in acetonitrile		
Gradient elution	Time (min)	A (%)	B (%)
	0	80	20
	40	40	60
	50	5	95
	55	5	95
	60	80	20
	70	80	20

PDA; photo-diode array detector

Table S2

System suitability for simultaneous analysis of the nine target components by HPLC–PDA.

Analyte[1]	Parameter[2]				
	k′	*α*	*N*/m	*Rs*	*S*
1	2.10	1.14	69,811	3.32	1.13
2	2.39	1.09	104,825	2.51	1.09
3	2.60	1.09	125,824	2.51	1.11
4	2.88	1.11	164,127	3.51	1.08
5	3.23	1.51	202,386	21.35	1.08
6	4.87	1.13	351,906	7.70	1.06
7	5.50	1.12	390,245	6.71	1.11
8	6.17	1.20	232,020	12.02	0.96
9	7.42	1.20	570,688	12.02	1.09
10	8.91	1.07	675,178	6.72	1.09
11	9.55	1.07	808,899	6.72	1.05
12	10.97	1.08	971,019	8.82	1.17
13	11.87	1.08	911,915	8.82	1.03

[1] Liquiritin apioside (1), liquiritin (2), 4-hydroxycinnamic acid (3), narirutin (4), naringin (5), ononin (6), baicalin (7), poncirin (8), wogonoside (9), baicalein (10), isoliquiritigenin (11), glycyrrhizin (12), and wogonin (13).

[2] Parameters: *k′*; retention factor, *α*; selectivity factor, *N*/m; theoretical plate number per meter [5.54 × (retention time / peak width at half peak height)^2] / column length (m), *Rs*; resolution, and *S*; symmetry factor

Table S3

Repeatability for retention time and peak area of the 13 marker compounds by HPLC–PDA assay.

Analyte[1]	Retention time (min)			Peak area (mAU)		
	Mean	SD × 10^{-1}	RSD (%)	Mean	SD	RSD (%)
1	7.93	0.03	0.04	1,426,119.38	6647.79	0.47
2	8.68	0.04	0.04	1,690,454.25	8256.64	0.49
3	9.21	0.04	0.04	1,147,345.88	5534.01	0.48
4	9.90	0.05	0.05	796,109.88	4339.55	0.55
5	10.80	0.05	0.05	690,824.38	3260.71	0.47
6	15.00	0.06	0.04	896,733.63	4460.46	0.50
7	16.28	0.56	0.34	921,160.75	5224.84	0.57
8	18.31	0.04	0.02	893,069.75	4552.56	0.51
9	21.15	0.58	0.28	1,018,057.75	4900.67	0.48
10	25.32	0.08	0.03	1,351,802.50	10167.50	0.75
11	26.93	0.04	0.02	2,433,658.75	11867.85	0.49
12	29.94	0.83	0.28	1,382,704.50	6369.07	0.46
13	32.87	0.09	0.03	1,773,983.38	9354.70	0.53

[1] Liquiritin apioside (1), liquiritin (2), 4-hydroxycinnamic acid (3), narirutin (4), naringin (5), ononin (6), baicalin (7), poncirin (8), wogonoside (9), baicalein (10), isoliquiritigenin (11), glycyrrhizin (12), and wogonin (13).

Table S4

Composition of and information on Yeokwisan.

Latin name	Scientific name	Family	Using part	Extraction solvent	Yield (%)	Mixed amount (mg)	Mixed ratio (%)
Glycyrrhizae Radix et Rhizoma	*Glycyrrhiza uralensis* Fisch.	Leguminosae	Root and Rhizome	30% Ethanol	24.7	55.9	16.9
Massa Medicata Fermentata	-	-	Yeast	30% Ethanol	9.3	27.8	8.4
Phyllostachyos Caulis in Taeniam	*Phyllostachys bambusoides* Sieb. et Zucc.	Gramineae	Middle of a stem	30% Ethanol	5.6	16.7	5.1
Ponciri Fructus Immaturus	*Poncirus trifoliata* (L.) Raf.	Rutaceae	Immature fruit	30% Ethanol	19.4	58.1	17.5
Scutellariae Radix	*Scutellaria baicalensis* Georgi	Labiatae	Root	30% Ethanol	50.2	150.5	45.4
Ostreae Testa	*Ostrea gigas* Thunb.	Ostreidae	Shell	Water	1.9	22.2	6.7
			Total			331.2	100.0

Table S5

Information on the 13 reference standard compounds selected as marker compounds for quality control of YWS.

Analyte[1]	Purity (%)	Molecular formula	CAS No.	PubChem CID	Catalog No.	Maker
1	99.6	$C_{26}H_{30}O_{13}$	74639-14-8	10076238	DR19690	Shanghai Sunny Biotech
2	99.6	$C_{21}H_{22}O_9$	551-15-5	503737	BP0874	Biopurify Phytochemicals
3	99.2	$C_9H_8O_3$	501-98-4	637542	082-06521	Fujifilm Wako Pure Chemical
4	99.5	$C_{27}H_{32}O_{14}$	14259-46-2	442431	BP0985	Biopurify Phytochemicals
5	98.0	$C_{27}H_{32}O_{14}$	10236-47-2	442428	CFN99555	Wuhan ChemFaces Biochemical
6	98.5	$C_{22}H_{22}O_9$	486-62-4	442813	BP1031	Biopurify Phytochemicals
7	98.5	$C_{21}H_{18}O_{11}$	21967-41-9	64982	DR10626	Shanghai Sunny Biotech
8	98.9	$C_{28}H_{34}O_{14}$	14941-08-3	442456	DR11185	Shanghai Sunny Biotech
9	98.9	$C_{22}H_{20}O_{11}$	51059-44-0	3084961	DR10630	Shanghai Sunny Biotech
10	99.4	$C_{15}H_{10}O_5$	491-67-8	5281605	DR10625	Shanghai Sunny Biotech
11	99.8	$C_{15}H_{12}O_4$	961-29-5	638278	TB0235-0500	ChemNorm Biotech
12	99.1	$C_{42}H_{62}O_{16}$	1405-86-3	14982	BP0682	Biopurify Phytochemicals
13	98.9	$C_{16}H_{12}O_5$	632-85-9	5281703	CFN97089	Wuhan ChemFaces Biochemical

[1] Liquiritin apioside (1), liquiritin (2), 4-hydroxycinnamic acid (3), narirutin (4), naringin (5), ononin (6), baicalin (7), poncirin (8), wogonoside (9), baicalein (10), isoliquiritigenin (11), glycyrrhizin (12), and wogonin (13).

Table S6

UPLC–MS/MS MRM conditions for simultaneous quantitative analysis of the 13 marker compounds in YWS sample.

UPLC conditions		MS conditions	
UPLC system	Acquity UPLC H-Class	MS system	TQ-S micro
Column	Gemini® C_{18} column (2.0 mm × 150 mm, 3 µm)	MS software	MassLynx v4.2
Column temp.	40 °C	Ion source	ESI^+ or ESI^-
Sample temp.	5 °C	Acquisition mode	MRM
Injection volume	2.0 µL	Capillary voltage	3.3 kV
Flow rate	0.3 mL/min	Cone gas flow	80 L/h
Mobile phase A	0.1% (v/v) formic acid in deionized water	Desolvation gas flow	600 L/h
Mobile phase B	0.1% (v/v) formic acid in acetonitrile	Desolvation temp.	300 °C
Gradient	see below	Source temp.	150 °C

Gradient:

Time (min)	A (%)	B (%)
Initial	82	18
6.5	50	50
9.0	50	50
9.1	82	18
12.0	82	18

ESI; electrospray ionization, MRM; multiple reaction monitoring

Table S7

Repeatability for retention time and peak area of the 13 marker compounds by UPLC–MS/MS MRM assay.

Analyte[1]	Retention time (min)			Peak area (mAU)		
	Mean	SD	RSD (%)	Mean	SD	RSD (%)
1	3.79	0.01	0.30	16836.02	230.07	1.37
2	3.99	0.01	0.29	3200.75	136.04	4.25
3	4.19	0.01	0.21	574.78	44.69	7.77
4	4.25	0.01	0.27	12199.21	1354.02	11.10
5	4.46	0.01	0.20	20437.71	1482.58	7.25
6	5.39	0.01	0.13	102857.19	1024.40	1.00
7	5.81	0.01	0.19	3921505.64	30532.62	0.78
8	5.88	0.01	0.19	20603.12	1771.24	8.60
9	6.91	0.01	0.14	1985701.38	6986.32	0.35
10	7.91	0.01	0.14	86777.98	1183.99	1.36
11	8.42	0.01	0.09	2730.43	188.81	6.92
12	8.71	0.01	0.11	123513.33	8598.93	6.96
13	9.38	0.01	0.07	159047.31	884.60	0.56

[1] Liquiritin apioside (1), liquiritin (2), 4-hydroxycinnamic acid (3), narirutin (4), naringin (5), ononin (6), baicalin (7), poncirin (8), wogonoside (9), baicalein (10), isoliquiritigenin (11), glycyrrhizin (12), and wogonin (13).

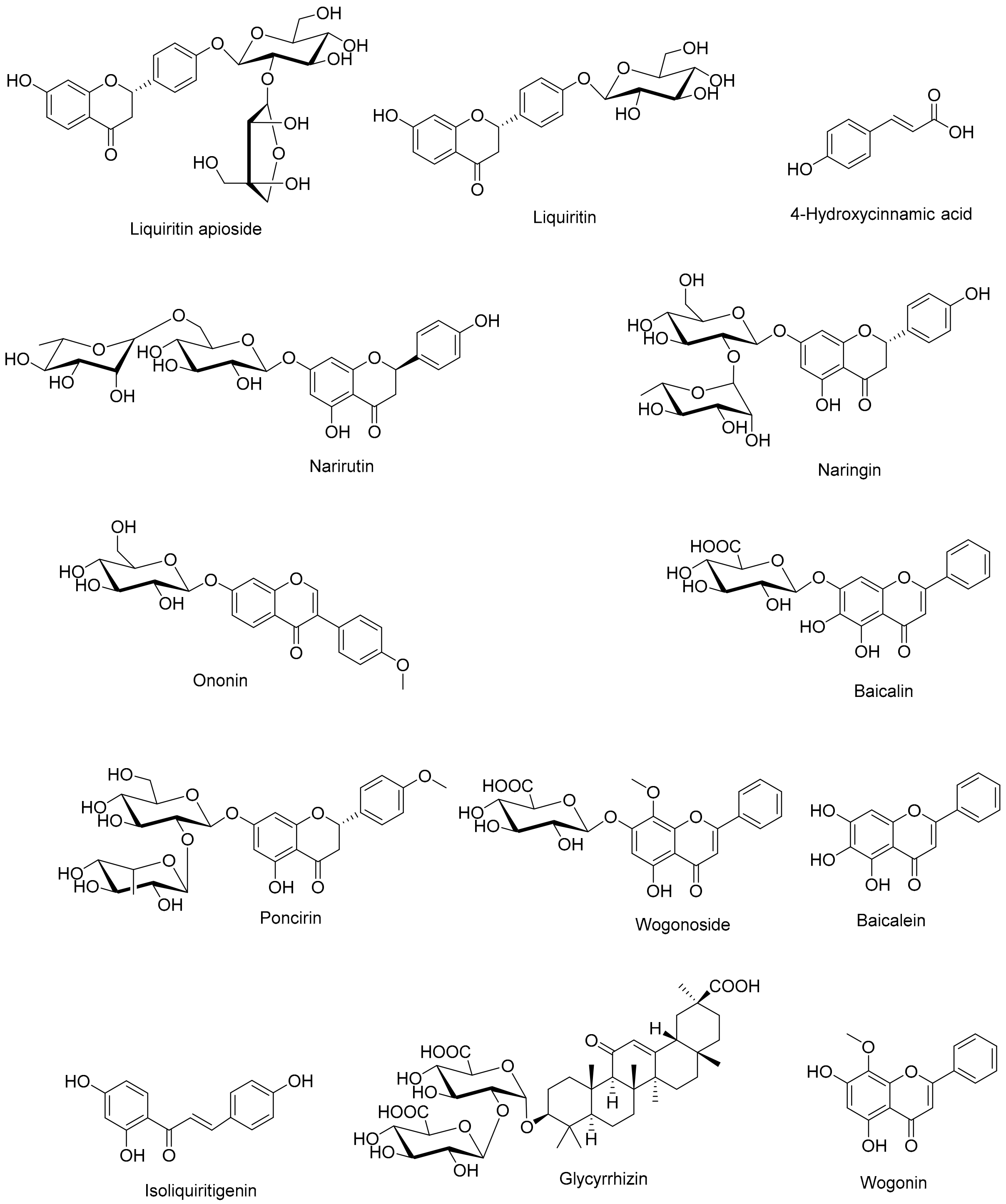

Figure S1. Chemical structures of the 13 reference standard compounds selected as marker compounds for quality control of YWS.

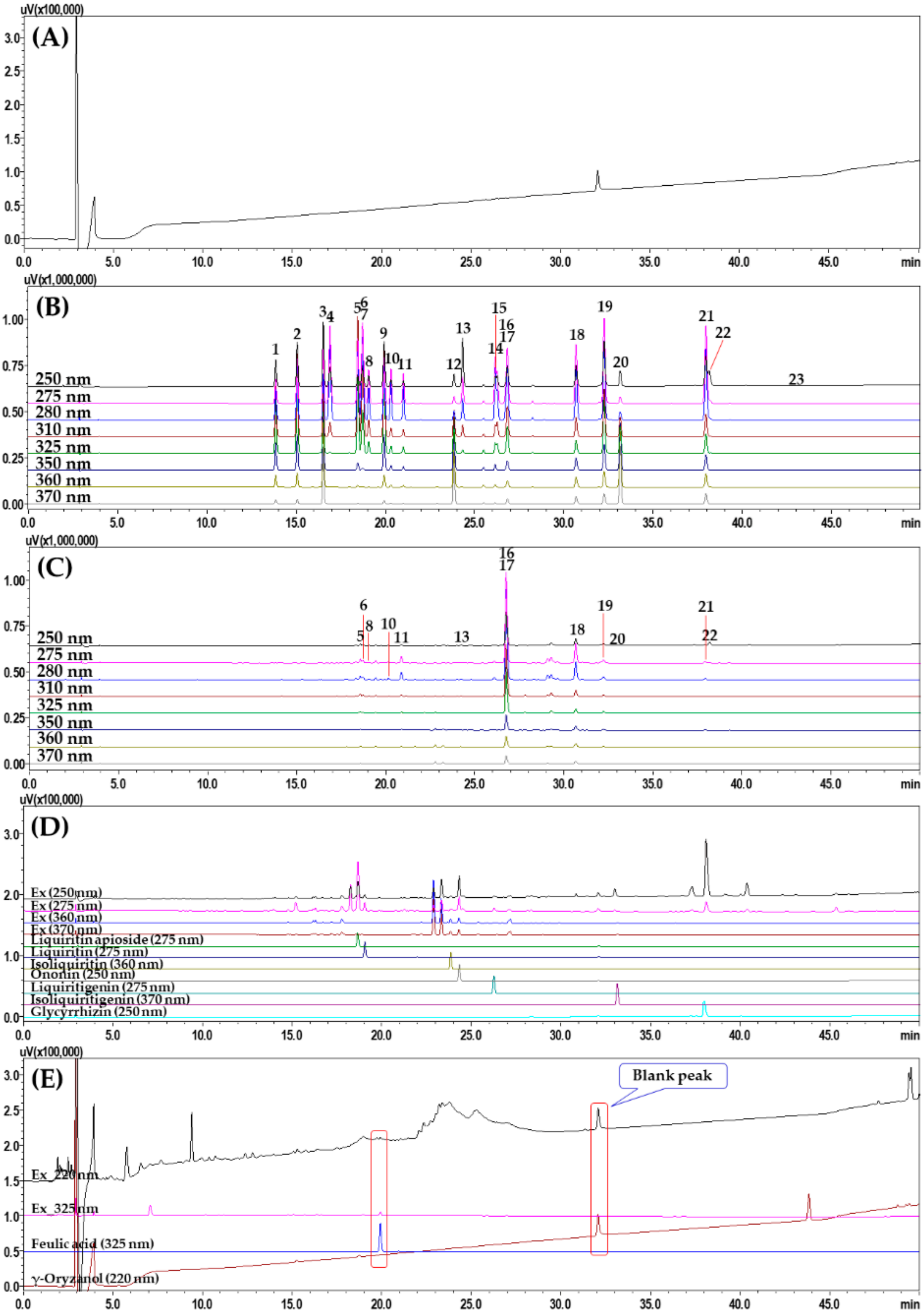
(A)
(B)
250 nm
275 nm
280 nm
310 nm
325 nm
350 nm
360 nm
370 nm
(C)
(D)
Ex (250 nm)
Ex (275 nm)
Ex (360 nm)
Ex (370 nm)
Liquiritin apioside (275 nm)
Liquiritin (275 nm)
Isoliquiritin (360 nm)
Ononin (250 nm)
Liquiritigenin (275 nm)
Isoliquiritigenin (370 nm)
Glycyrrhizin (250 nm)
(E)
Blank peak
Ex_220 nm
Ex_325 nm
Feulic acid (325 nm)
γ-Oryzanol (220 nm)
min

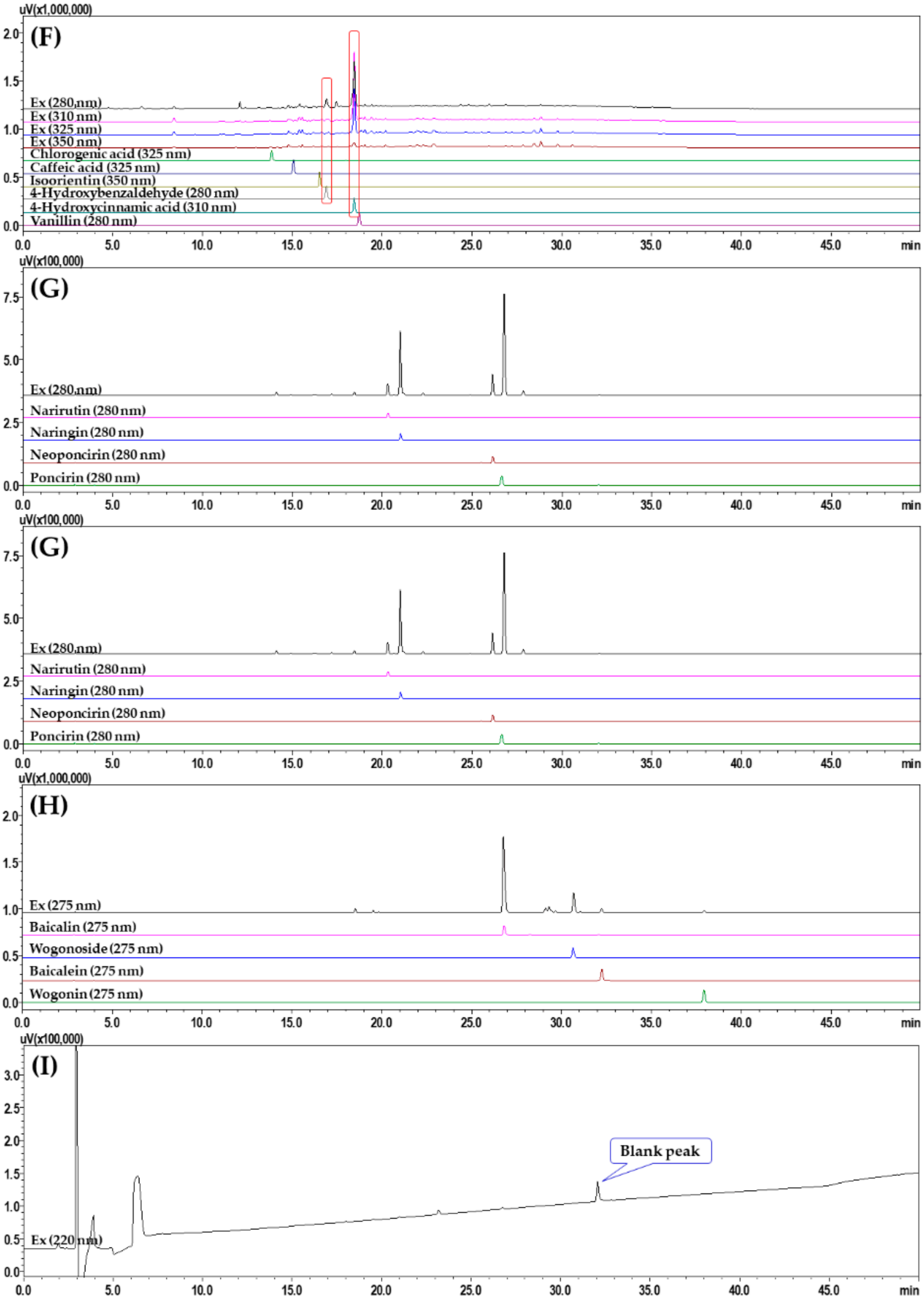

(F)
uV(x1,000,000)
Ex (280 nm)
Ex (310 nm)
Ex (325 nm)
Ex (350 nm)
Chlorogenic acid (325 nm)
Caffeic acid (325 nm)
Isoorientin (350 nm)
4-Hydroxybenzaldehyde (280 nm)
4-Hydroxycinnamic acid (310 nm)
Vanillin (280 nm)
min
(G)
uV(x100,000)
Ex (280 nm)
Narirutin (280 nm)
Naringin (280 nm)
Neoponcirin (280 nm)
Poncirin (280 nm)
min
(G)
uV(x100,000)
Ex (280 nm)
Narirutin (280 nm)
Naringin (280 nm)
Neoponcirin (280 nm)
Poncirin (280 nm)
min
(H)
uV(x1,000,000)
Ex (275 nm)
Baicalin (275 nm)
Wogonoside (275 nm)
Baicalein (275 nm)
Wogonin (275 nm)
min
(I)
uV(x100,000)
Ex (220 nm)
Blank peak
min

Figure S2. HPLC–PDA chromatograms investigated for the selection of marker compounds. A; Blank sample. B; Mixed 23 standard compounds. C; 70% methanol solution of lyophilized YWS sample. D; Glycyrrhizae Radix et Rhizoma extract. E; Massa Medicata Fermentata extract. F; Phyllostachyos Caulis in Taeniam extract. G; Ponciri Fructus Immaturus extract. H; Scutellariae Radix extract. and I; Ostreae Testa extract. Chlorogenic acid (1), caffeic acid (2), isoorientin (3), 4-hydroxybenzaldehyde (4), 4-hydroxycinnamic acid (5), liquiritin apioside (6), vanillin (7), liquiritin (8), ferulic acid (9), narirutin (10), naringin (11), isoliquiritin (12), ononin (13), neoponcirin (14), liquiritigenin (15), poncirin (16), baicalin (17), wogonoside (18), baicalein (19), isoliquiritigenin (20), wogonin (21), glycyrrhizin (22), and γ-oryzanol (23). Twenty-three components were separated using gradient elution of a 0.1% aqueous formic acid (solvent A)–0.1% formic acid in acetonitrile (solvent B) mobile phase system on a SunFire™ C_{18} column maintained at 40 °C. The gradient elution program for the mobile phase is as follows: 5% B (0 min), 60% B (40 min), 100% B (50 min, hold for 5 min), and 5% B (60 min).

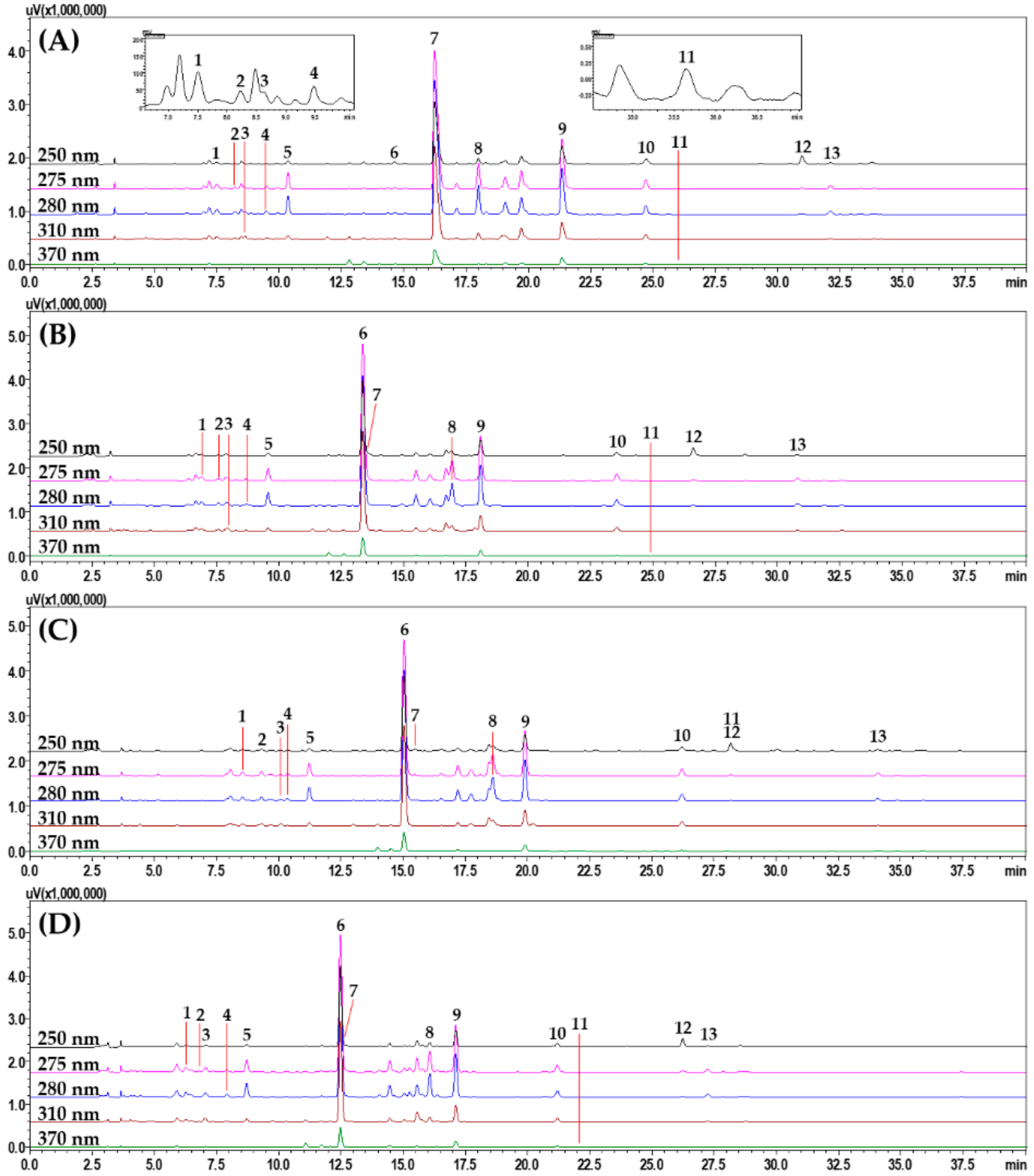

Figure S3. Comparison of HPLC–PDA chromatograms according to column manufacturer. A; SunFire™ C_{18} column. Liquiritin apioside (1), liquiritin (2), 4-hydroxycinnamic acid (3), narirutin (4), naringin (5), ononin (6), baicalin (7), poncirin (8), wogonoside (9), baicalein (10), isoliquiritigenin (11), glycyrrhizin (12), and wogonin (13). B; Capcell Pak UG80 C_{18} column. C; YMC-Triart C_{18} column. D; Hypersil GOLD C_{18} column. Liquiritin apioside (1), liquiritin (2), 4-hydroxycinnamic acid (3), narirutin (4), naringin (5), baicalin (6), ononin (7), poncirin (8), wogonoside (9), baicalein (10), isoliquiritigenin (11), glycyrrhizin (12), and wogonin (13).

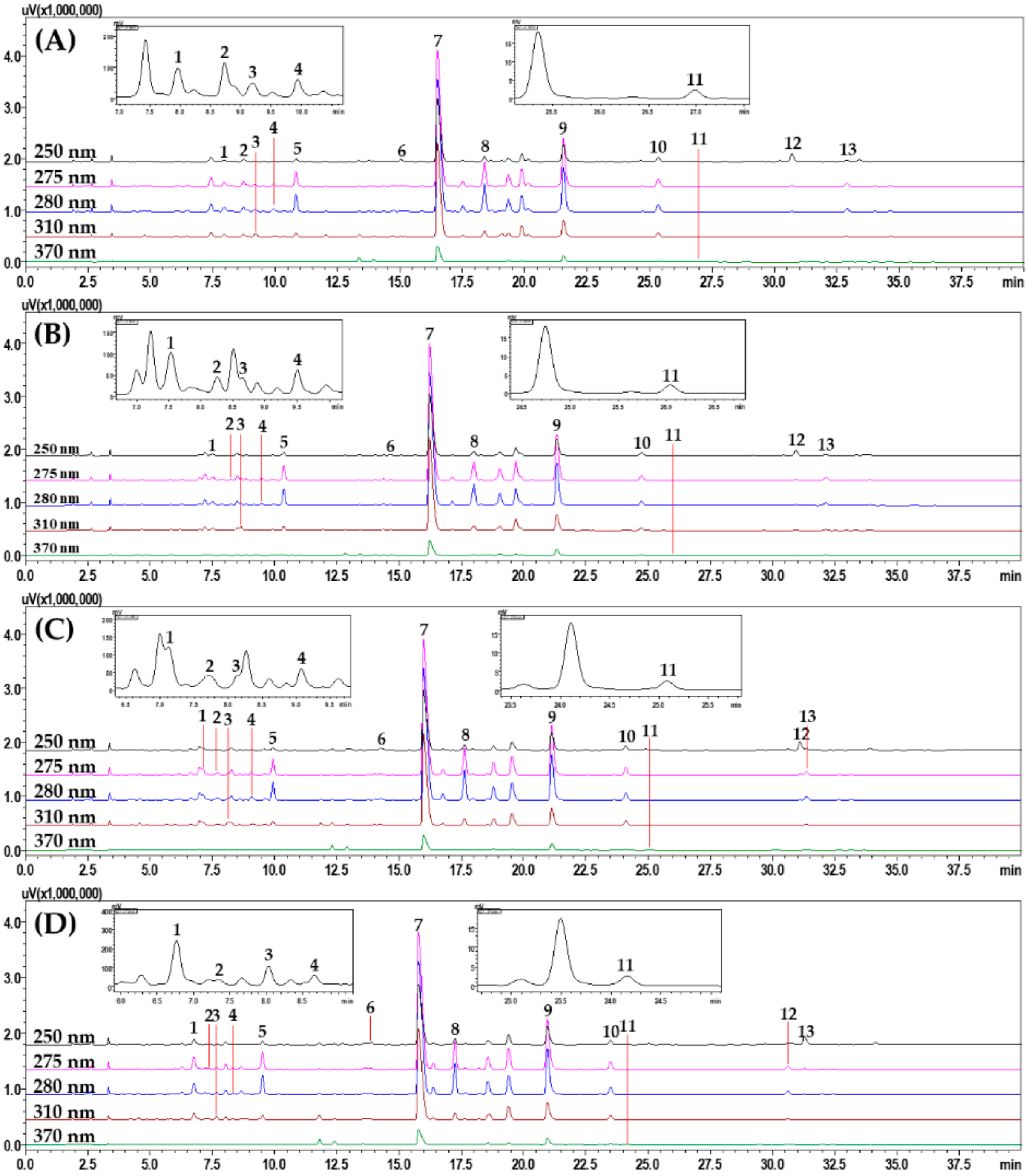

Figure S4. Comparison of HPLC–PDA chromatograms according to column temperatures of the 13 selected marker compounds. 35 °C (A), 40 °C (B), 45 °C (C), and 50 °C (D). Liquiritin apioside (1), liquiritin (2), 4-hydroxycinnamic acid (3), narirutin (4), naringin (5), ononin (6), baicalin (7), poncirin (8), wogonoside (9), baicalein (10), isoliquiritigenin (11), glycyrrhizin (12), and wogonin (13).

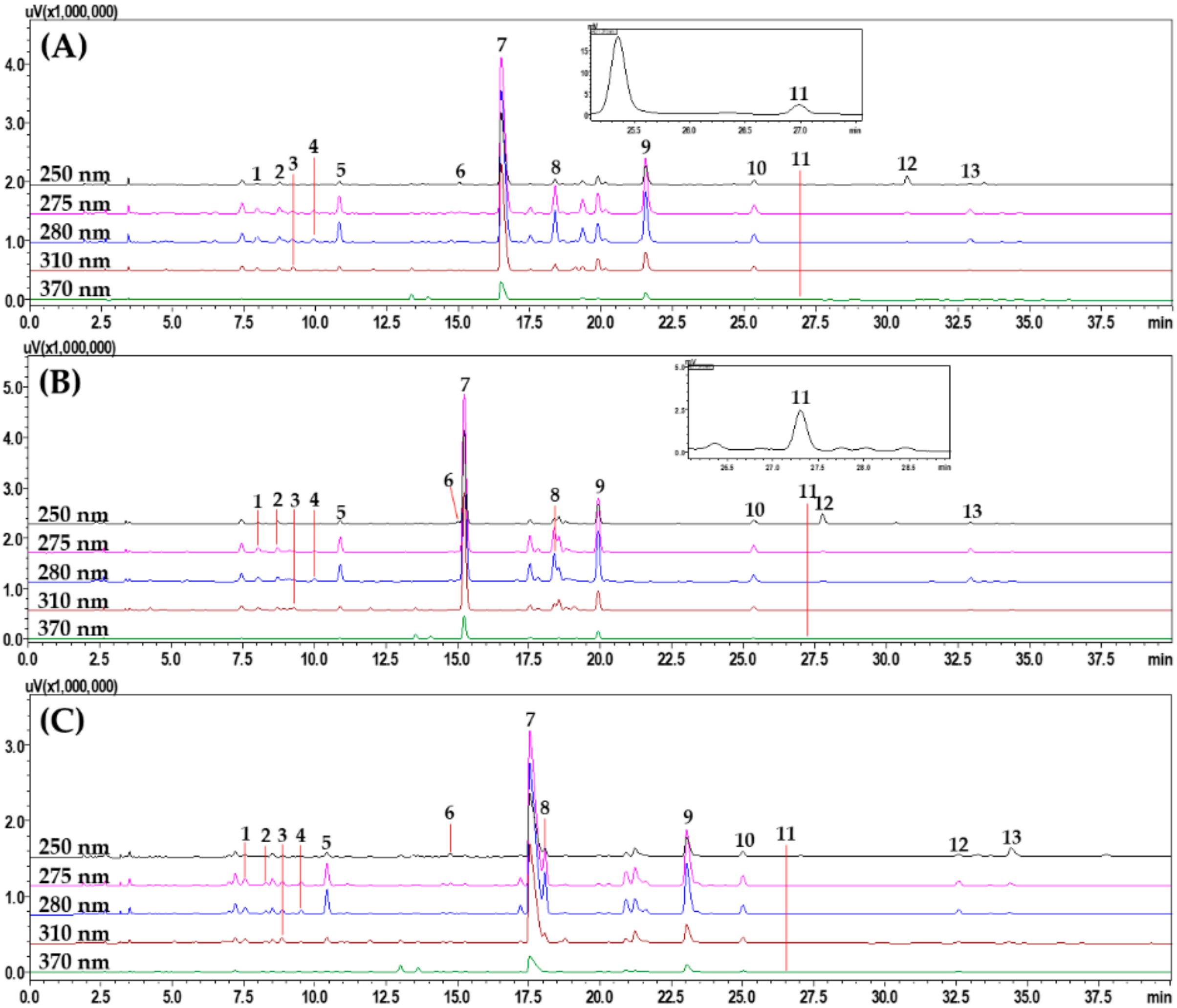

Figure S5. Comparison of HPLC–PDA chromatograms according to the type of acid for the 13 selected marker compounds. 0.1% (v/v) Formic acid (A), 0.1% (v/v) trifluoroacetic acid (B), and 1.0% (v/v) acetic acid (C). Liquiritin apioside (1), liquiritin (2), 4-hydroxycinnamic acid (3), narirutin (4), naringin (5), ononin (6), baicalin (7), poncirin (8), wogonoside (9), baicalein (10), isoliquiritigenin (11), glycyrrhizin (12), and wogonin (13).

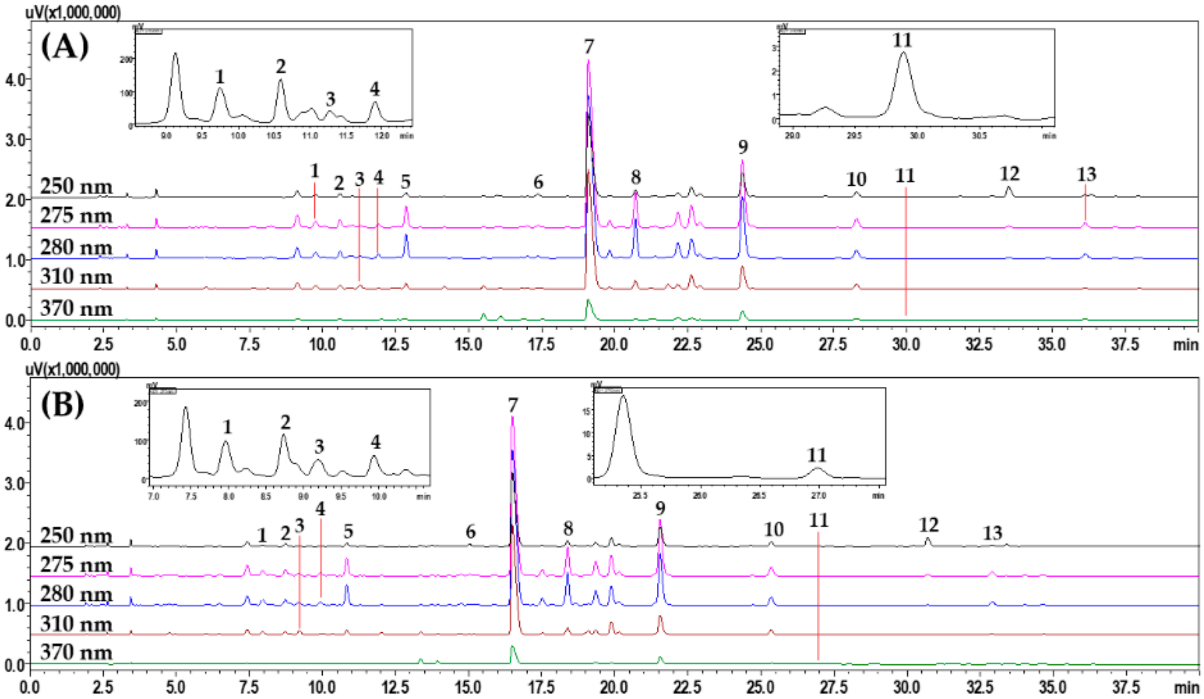

Figure S6. Comparison of HPLC–PDA chromatograms according to the flow rate for the 13 selected marker compounds. 0.8 mL/min (A) and 1.0 mL/min (B). Liquiritin apioside (1), liquiritin (2), 4-hydroxycinnamic acid (3), narirutin (4), naringin (5), ononin (6), baicalin (7), poncirin (8), wogonoside (9), baicalein (10), isoliquiritigenin (11), glycyrrhizin (12), and wogonin (13).

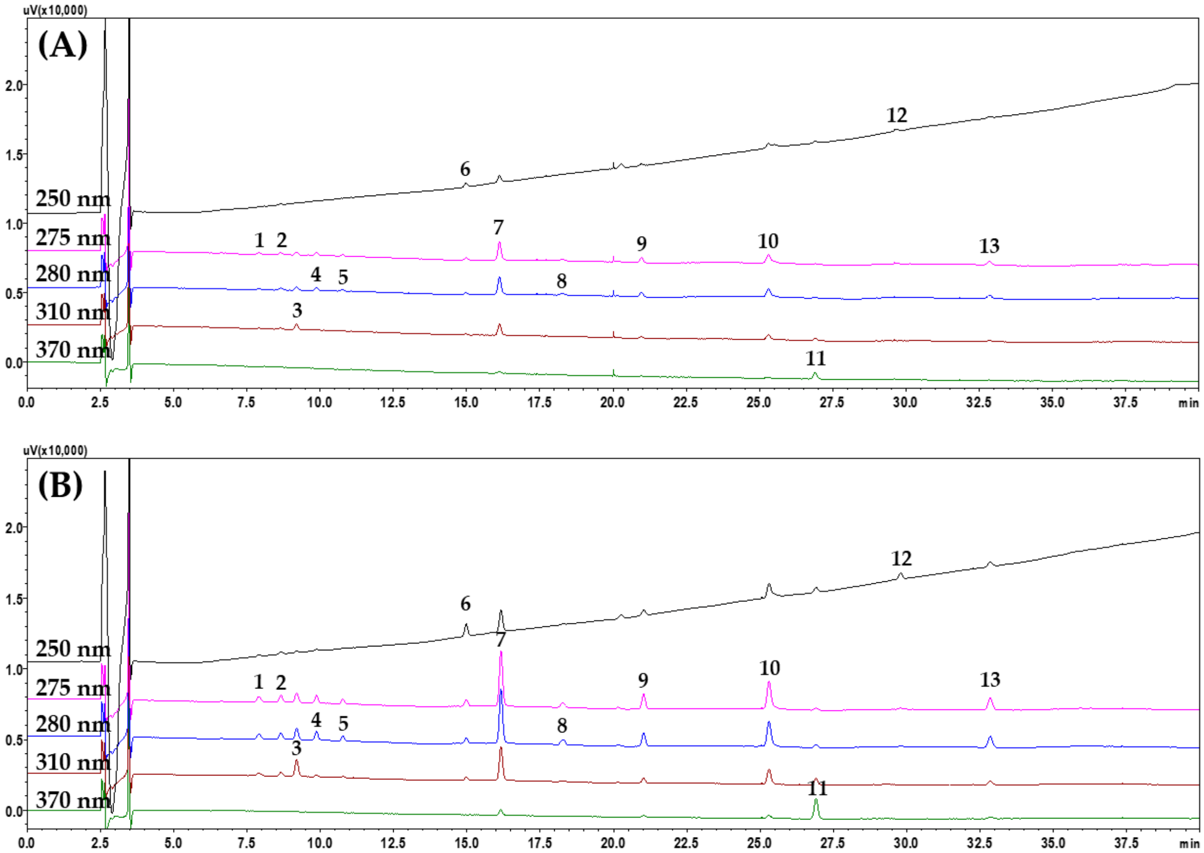

Figure S7. HPLC chromatograms at LOD (A) and LOQ (B) concentrations. Liquiritin apioside (1), liquiritin (2), 4-hydroxycinnamic acid (3), narirutin (4), naringin (5), ononin (6), baicalin (7), poncirin (8), wogonoside (9), baicalein (10), isoliquiritigenin (11), glycyrrhizin (12), and wogonin (13).

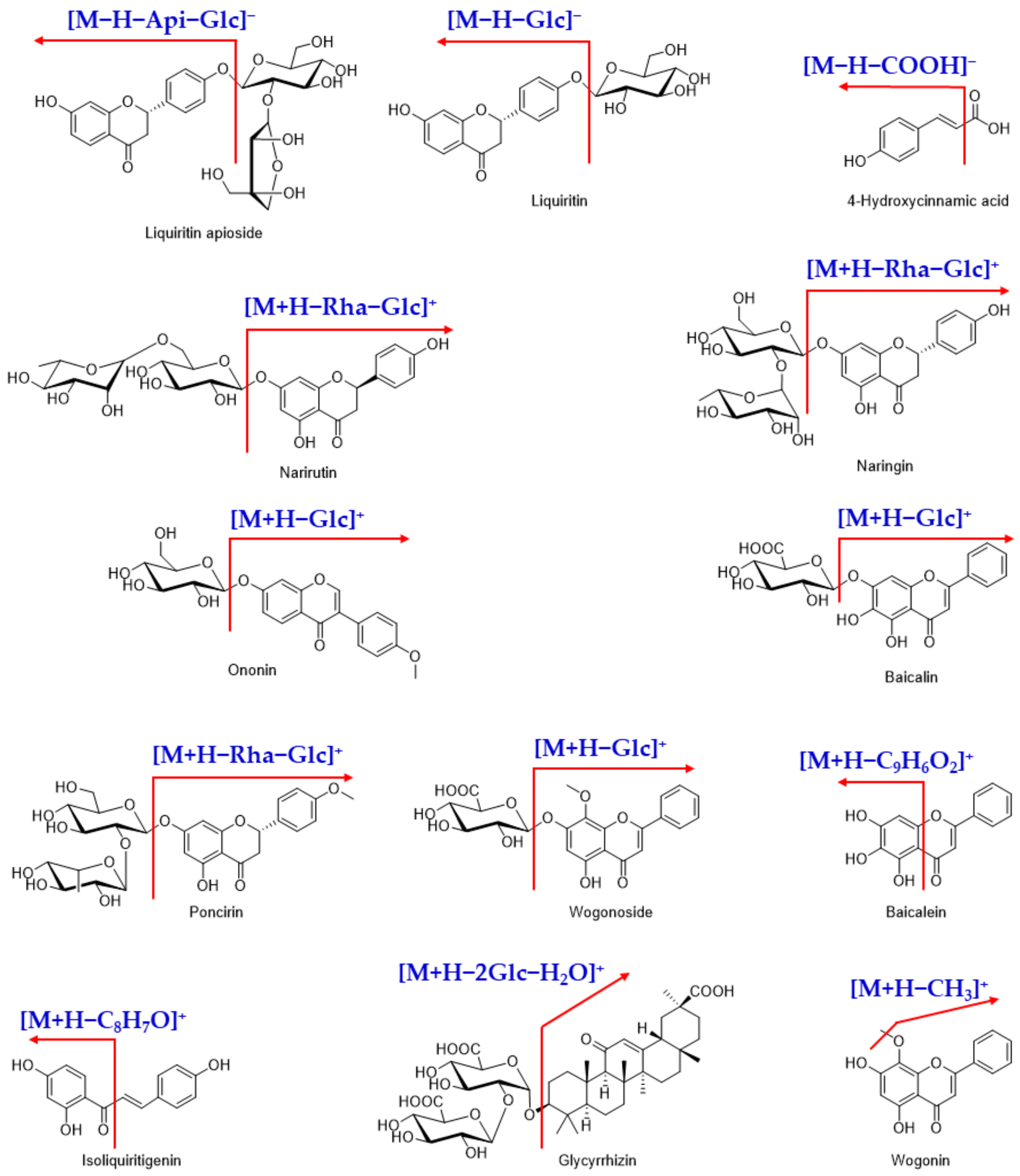

Figure S8. Mass fragmentation of each marker compound for simultaneous analysis.

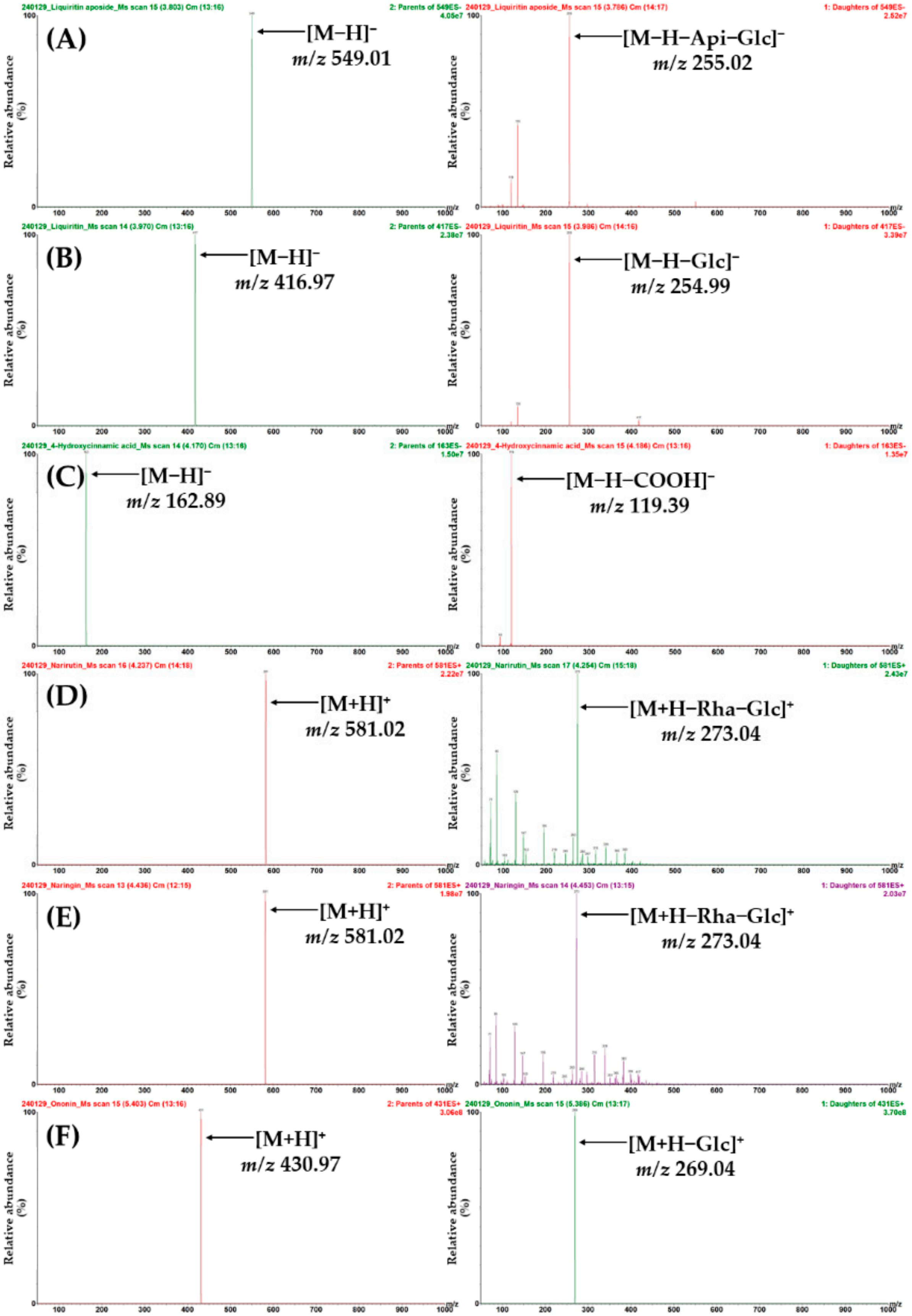

(A)
[M−H]⁻
m/z 549.01
[M−H−Api−Glc]⁻
m/z 255.02
(B)
[M−H]⁻
m/z 416.97
[M−H−Glc]⁻
m/z 254.99
(C)
[M−H]⁻
m/z 162.89
[M−H−COOH]⁻
m/z 119.39
(D)
[M+H]⁺
m/z 581.02
[M+H−Rha−Glc]⁺
m/z 273.04
(E)
[M+H]⁺
m/z 581.02
[M+H−Rha−Glc]⁺
m/z 273.04
(F)
[M+H]⁺
m/z 430.97
[M+H−Glc]⁺
m/z 269.04
Relative abundance (%)

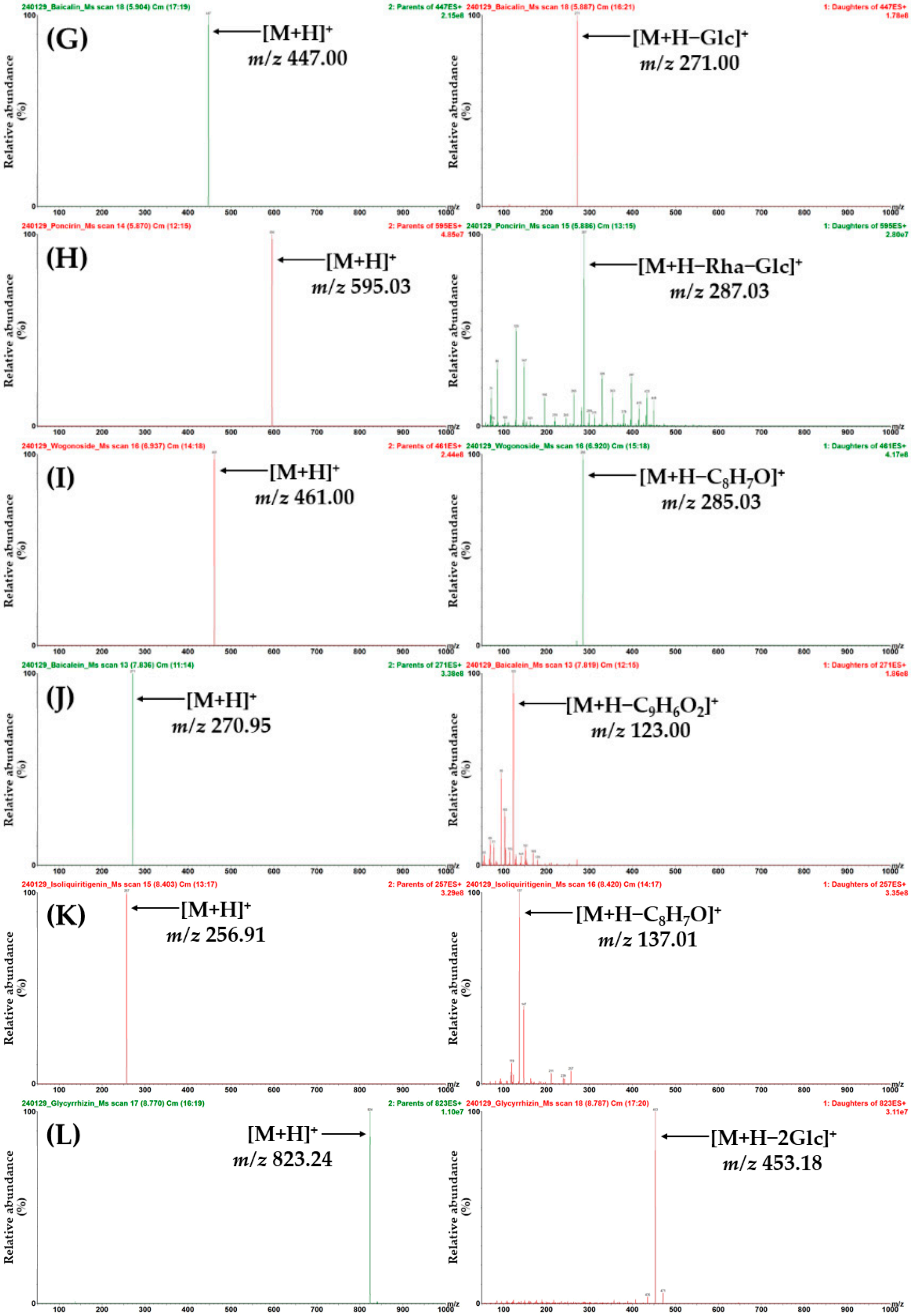

240129_Baicalin_Ms scan 18 (5.904) Cm (17:19)
2: Parents of 447ES+
2.15e8
(G)
[M+H]+
m/z 447.00
240129_Baicalin_Ms scan 18 (5.887) Cm (16:21)
1: Daughters of 447ES+
1.78e8
[M+H−Glc]+
m/z 271.00
240129_Poncirin_Ms scan 14 (5.870) Cm (12:15)
2: Parents of 595ES+
4.85e7
(H)
[M+H]+
m/z 595.03
240129_Poncirin_Ms scan 15 (5.886) Cm (13:15)
1: Daughters of 595ES+
2.80e7
[M+H−Rha−Glc]+
m/z 287.03
240129_Wogonoside_Ms scan 16 (6.937) Cm (14:18)
2: Parents of 461ES+
2.44e8
(I)
[M+H]+
m/z 461.00
240129_Wogonoside_Ms scan 16 (6.920) Cm (15:18)
1: Daughters of 461ES+
4.17e8
[M+H−C8H7O]+
m/z 285.03
240129_Baicalein_Ms scan 13 (7.836) Cm (11:14)
2: Parents of 271ES+
3.38e8
(J)
[M+H]+
m/z 270.95
240129_Baicalein_Ms scan 13 (7.819) Cm (12:15)
1: Daughters of 271ES+
1.86e8
[M+H−C9H6O2]+
m/z 123.00
240129_Isoliquiritigenin_Ms scan 15 (8.403) Cm (13:17)
2: Parents of 257ES+
3.29e8
(K)
[M+H]+
m/z 256.91
240129_Isoliquiritigenin_Ms scan 16 (8.420) Cm (14:17)
1: Daughters of 257ES+
3.35e8
[M+H−C8H7O]+
m/z 137.01
240129_Glycyrrhizin_Ms scan 17 (8.770) Cm (16:19)
2: Parents of 823ES+
1.10e7
(L)
[M+H]+
m/z 823.24
240129_Glycyrrhizin_Ms scan 18 (8.787) Cm (17:20)
1: Daughters of 823ES+
3.11e7
[M+H−2Glc]+
m/z 453.18
Relative abundance (%)
m/z

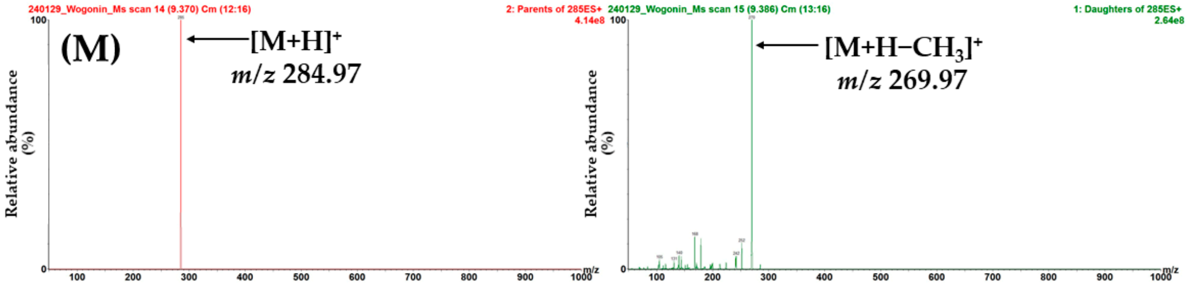

Figure S9. Precursor ion (Q1; left) and product ion (Q3; right) peaks for each marker compound. Liquiritin apioside (1), liquiritin (2), 4-hydroxycinnamic acid (3), narirutin (4), naringin (5), ononin (6), baicalin (7), poncirin (8), wogonoside (9), baicalein (10), isoliquiritigenin (11), glycyrrhizin (12), and wogonin (13).

(A)

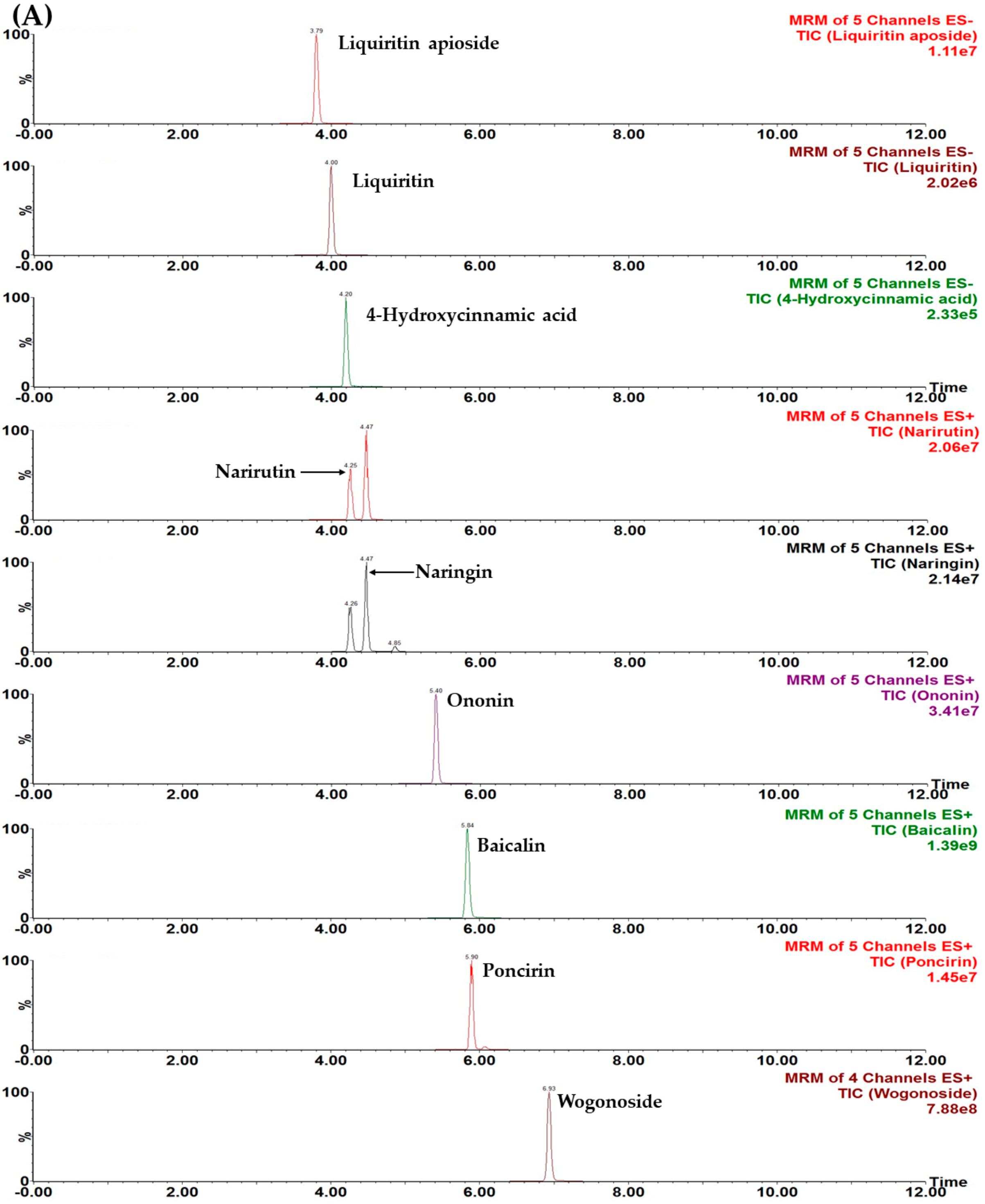
MRM of 5 Channels ES-
TIC (Liquiritin aposide)
1.11e7
Liquiritin apioside
MRM of 5 Channels ES-
TIC (Liquiritin)
2.02e6
Liquiritin
MRM of 5 Channels ES-
TIC (4-Hydroxycinnamic acid)
2.33e5
4-Hydroxycinnamic acid
MRM of 5 Channels ES+
TIC (Narirutin)
2.06e7
Narirutin
MRM of 5 Channels ES+
TIC (Naringin)
2.14e7
Naringin
MRM of 5 Channels ES+
TIC (Ononin)
3.41e7
Ononin
MRM of 5 Channels ES+
TIC (Baicalin)
1.39e9
Baicalin
MRM of 5 Channels ES+
TIC (Poncirin)
1.45e7
Poncirin
MRM of 4 Channels ES+
TIC (Wogonoside)
7.88e8
Wogonoside
Time
%
100
0
-0.00
2.00
4.00
6.00
8.00
10.00
12.00

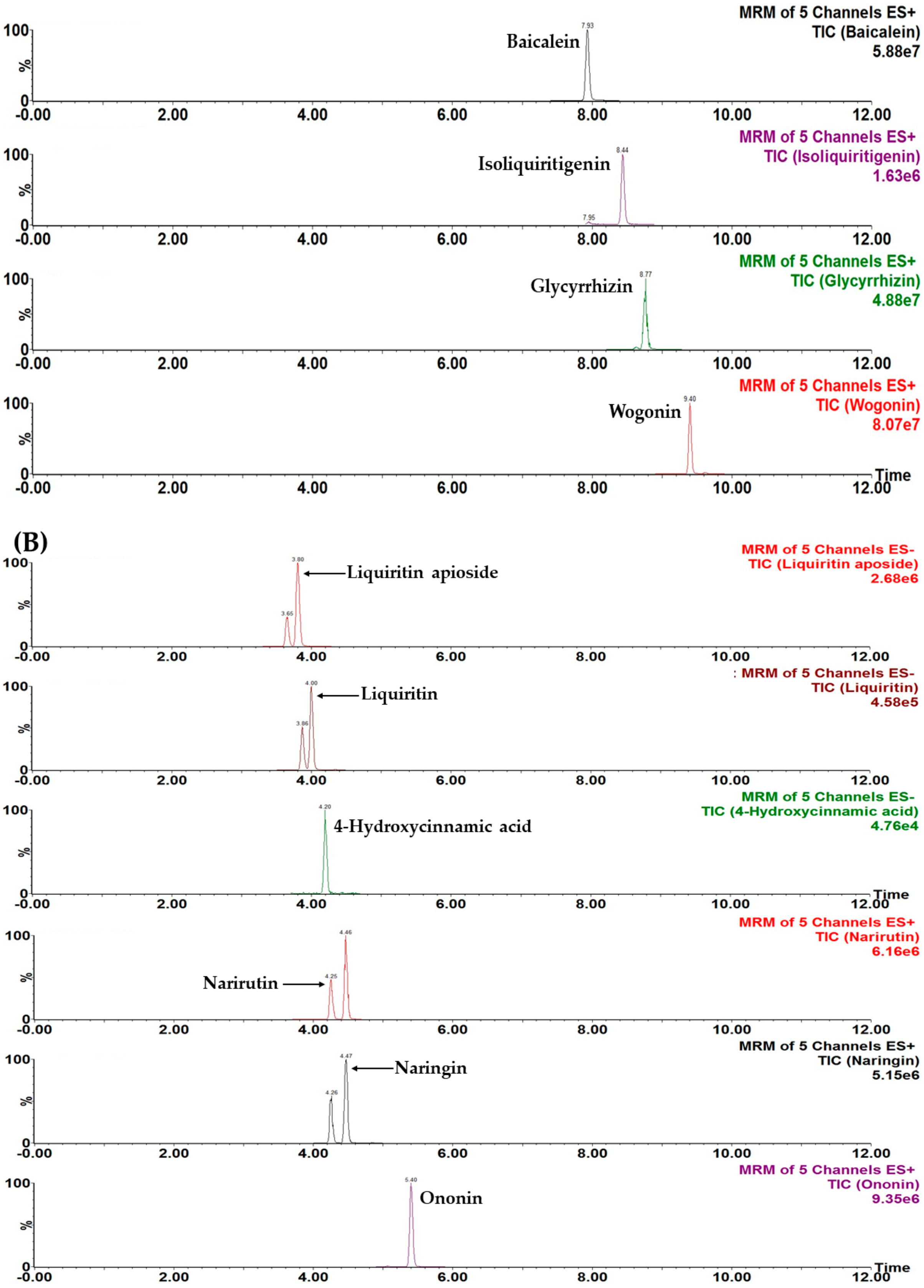

MRM of 5 Channels ES+
TIC (Baicalein)
5.88e7
Baicalein
MRM of 5 Channels ES+
TIC (Isoliquiritigenin)
1.63e6
Isoliquiritigenin
MRM of 5 Channels ES+
TIC (Glycyrrhizin)
4.88e7
Glycyrrhizin
MRM of 5 Channels ES+
TIC (Wogonin)
8.07e7
Wogonin
Time
(B)
MRM of 5 Channels ES-
TIC (Liquiritin aposide)
2.68e6
Liquiritin apioside
MRM of 5 Channels ES-
TIC (Liquiritin)
4.58e5
Liquiritin
MRM of 5 Channels ES-
TIC (4-Hydroxycinnamic acid)
4.76e4
4-Hydroxycinnamic acid
MRM of 5 Channels ES+
TIC (Narirutin)
6.16e6
Narirutin
MRM of 5 Channels ES+
TIC (Naringin)
5.15e6
Naringin
MRM of 5 Channels ES+
TIC (Ononin)
9.35e6
Ononin
Time

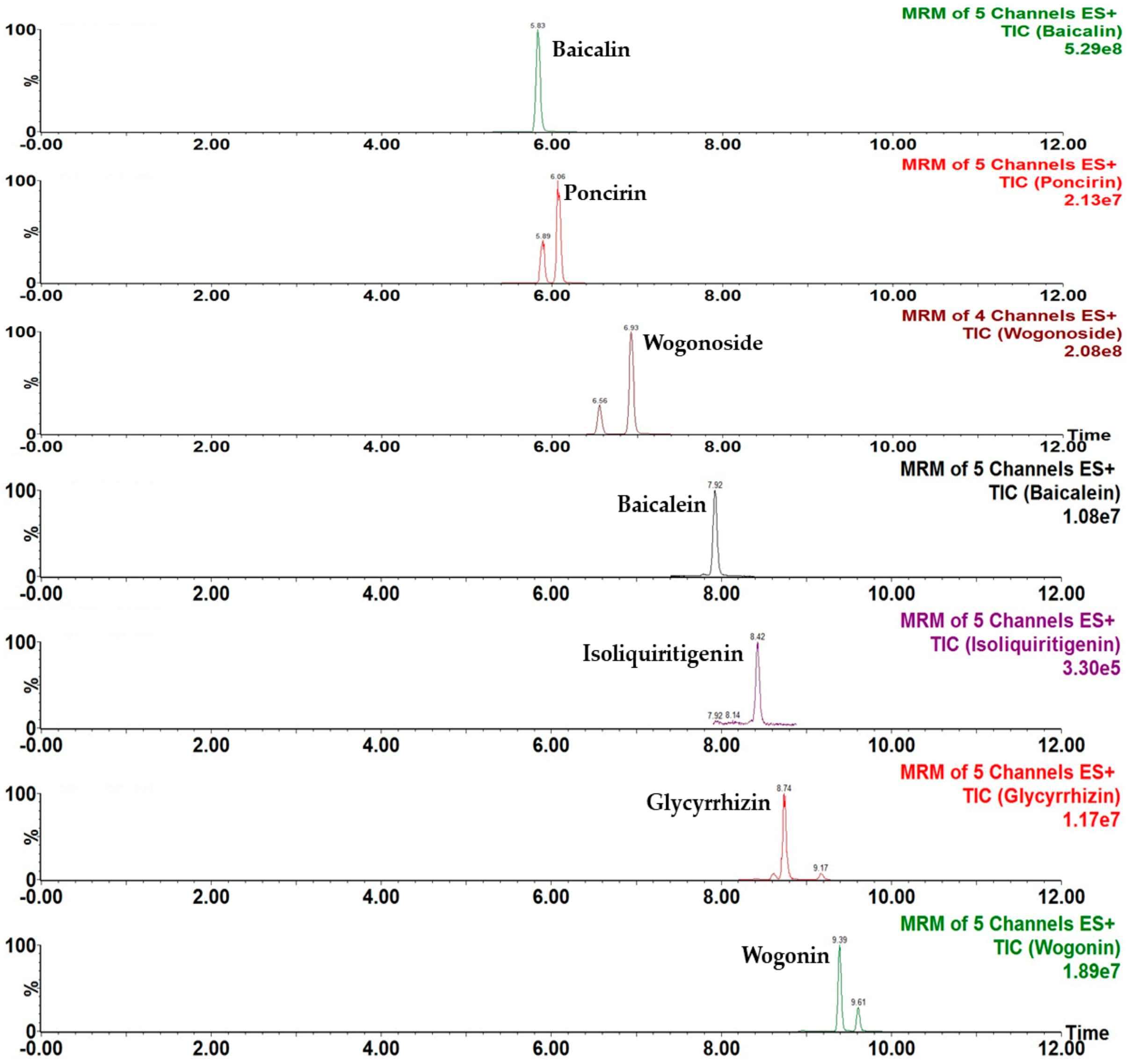

Figure S10. Extracted ion chromatograms of each reference standard compound (A) and the 13 marker compounds selected in YWS sample (B) by UPLC–MS/MS MRM method in negative and positive ion modes.
